# Deciphering the immunosuppressive tumor microenvironment in ALK- and EGFR-positive lung adenocarcinoma

**DOI:** 10.1007/s00262-021-02981-w

**Published:** 2021-06-14

**Authors:** Jan Budczies, Martina Kirchner, Klaus Kluck, Daniel Kazdal, Julia Glade, Michael Allgäuer, Mark Kriegsmann, Claus-Peter Heußel, Felix J. Herth, Hauke Winter, Michael Meister, Thomas Muley, Torsten Goldmann, Stefan Fröhling, Martin Wermke, Cornelius F. Waller, Amanda Tufman, Martin Reck, Solange Peters, Peter Schirmacher, Michael Thomas, Petros Christopoulos, Albrecht Stenzinger

**Affiliations:** 1 Institute of Pathology, Heidelberg University Hospital, Im Neuenheimer Feld 224, Heidelberg, Germany; 2 German Cancer Consortium (DKTK) and German Cancer Research Center (DKFZ), Heidelberg, Germany; 3 Translational Lung Research Center Heidelberg (TLRC-H), Member of the German Center for Lung Research (DZL), Heidelberg, Germany; 4 Department of Diagnostic and Interventional Radiology With Nuclear Medicine, Thoraxklinik at Heidelberg University Hospital, Heidelberg, Germany; 5 Department of Diagnostic and Interventional Radiology, University Hospital, Heidelberg, Germany; 6 Department of Pneumology, Thoraxklinik at Heidelberg University Hospital, Heidelberg, Germany; 7 Department of Thoracic Surgery, Thoraxklinik at Heidelberg University Hospital, Heidelberg, Germany; 8 Translational Research Unit, Thoraxklinik at Heidelberg University Hospital, Heidelberg, Germany; 9 Pathology of the University Medical Center Schleswig-Holstein (UKSH), Campus Lübeck and the Research Center Borstel, Borstel, Germany; 10 Airway Research Center North (ARCN), Member of German Center of Lung Research (DZL), Giessen, Germany; 11 Department of Translational Oncology, National Center for Tumor Diseases (NCT), Heidelberg, Germany; 12 Department of Thoracic Oncology, Dresden University Hospital, Dresden, Germany; 13 Department of Haematology, Oncology and Stem Cell Transplantation, University Medical Centre Freiburg, Freiburg, Germany; 14 Division of Respiratory Medicine and Thoracic Oncology, Department of Internal Medicine V and Thoracic Oncology Centre Munich, Comprehensive Pneumology Center, Member of the German Center for Lung Research (DZL), University of Munich (LMU), Munich, Germany; 15 Department of Thoracic Oncology, Lung Clinic Grosshansdorf, Grosshansdorf, Germany; 16 Department of Oncology, Centre Hospitalier Universitaire Vaudois (CHUV), Lausanne University, Lausanne, Switzerland; 17 Department of Thoracic Oncology, Thoraxklinik At Heidelberg University Hospital, Heidelberg, Germany

**Keywords:** Lung adenocarcinoma, ALK fusion, EGFR mutation, Immunotherapy, Immune checkpoint blockade, Immunosuppression

## Abstract

**Introduction:**

The advent of immune checkpoint blockade (ICB) has led to significantly improved disease outcome in lung adenocarcinoma (ADC), but response of ALK/EGFR-positive tumors to immune therapy is limited. The underlying immune biology is incompletely understood.

**Methods:**

We performed comparative mRNA expression profiling of 31 ALK-positive, 40 EGFR-positive and 43 ALK/EGFR-negative lung ADC focused on immune gene expression. The presence and levels of tumor infiltration lymphocytes (TILs) as well as fourteen specific immune cell populations were estimated from the gene expression profiles.

**Results:**

While total TILs were not lower in ALK-positive and EGFR-positive tumors compared to ALK/EGFR-negative tumors, specific immunosuppressive characteristics were detected in both subgroups: In ALK-positive tumors, regulatory T cells were significantly higher compared to EGFR-positive (fold change: FC = 1.9, *p* = 0.0013) and ALK/EGFR-negative tumors (FC = 2.1, *p* = 0.00047). In EGFR-positive tumors, cytotoxic cells were significantly lower compared to ALK-positive (FC =  − 1.7, *p* = 0.016) and to ALK/EGFR-negative tumors (FC =  − 2.1, *p* = 2.0E-05). A total number of 289 genes, 40 part of cytokine–cytokine receptor signaling, were differentially expressed between the three subgroups. Among the latter, five genes were differently expressed in both ALK-positive and EGFR-positive tumors, while twelve genes showed differential expression solely in ALK-positive tumors and eleven genes solely in EGFR-positive tumors.

**Conclusion:**

Targeted gene expression profiling is a promising tool to read out tumor microenvironment characteristics from routine diagnostic lung cancer biopsies. Significant immune reactivity including specific immunosuppressive characteristics in ALK- and EGFR-positive lung ADC, but not a total absence of immune infiltration supports further clinical evaluation of immune-modulators as partners of ICB in such tumors.

**Supplementary Information:**

The online version contains supplementary material available at 10.1007/s00262-021-02981-w.

## Introduction

Non-small cell lung cancer (NSCLC) is the leading cause of cancer-related mortality worldwide [1]. Despite major advances in therapeutic options, the majority of patients are diagnosed at advanced stage with a median survival rate below 2 years. Genetic profiling is of key importance for treatment decision in NSCLC, since targeted therapies, mainly tyrosine kinase inhibition (TKI), have shown dramatically superior efficacy as comparted to standard of care chemotherapy in oncogene-addicted tumors [2]. EGFR mutations and ALK fusions represent the most frequent targetable alterations with a prevalence of, respectively, 10–20% and 3–5% in a Caucasian population with lung adenocarcinomas (ADC) [3].

While the advent of immune checkpoint blockade (ICB) led to significant advances in disease control and survival of driver-negative NSCLC, it quickly became apparent that ALK- and EGFR-positive tumors show limited response to ICB. For example, even despite encouraging early results from the Keynote-001 trail suggesting potential efficacy of pembrolizumab in TKI-naïve EGFR-mutated NSCLC, these were invalidated in a subsequent phase 2 trial [4, 5]. Similarly, Checkmate 012 [6], a phase 1 trial investigating first-line nivolumab monotherapy or in combination with standard therapies for NSCLC demonstrated no meaningful activity in EGFR-mutated tumors. The same trial also investigated the combination of nivolumab with platinum-based chemotherapy and showed reduced activity in EGFR-mutant compared to EGFR-wildtype tumors [7]. Furthermore, several clinical trials combining ICB and TKI resulted in significant toxicity without a signal of improved activity above TKI [7–9]. The only exception so far has been the IMpower150 study, which demonstrated a survival benefit for TKI-pretreated patients with ALK/EGFR-positive NSCLC when the PD-L1 inhibitor atezolizumab was combined with both the antiangiogenic agent bevacizumab and chemotherapy [10, 11].

The biological underpinnings for ICB primary resistance in patients with EGFR-/ALK-positive NSCLC are incompletely understood. Lower tumor mutational burden (TMB), for example, in tumors of never smokers [12, 13], a different nature of PD-L1 expression (intrinsic, induced by oncogenic signaling rather than tumor-infiltrating lymphocytes) [14, 15] and features of the tumor microenvironment (TME) [16–19], possibly influenced by MAPK-signaling [20], have all been suggested as potential causes. The TME is increasingly recognized as crucial parameter for the efficacy of immunotherapies in general, but biological data, especially for the less frequent ALK-positive tumors, are limited [21].

We employed the NanoString nCounter technology and the PanCancer Human IO 360 Panel to investigate the TME in 114 formalin-fixed and paraffin-embedded (FFPE) biopsies of clinically annotated ALK-positive, EGFR-positive and ALK/EGFR-negative advanced lung ADC. We also analyzed gene expression data of earlier stage, resectable tumors from the TCGA lung ADC cohort.

## Material and methods

### Study cohort

The retrospective study cohort included 31 ALK-positive, 40 EGFR-positive and 43 ALK/EGFR-negative lung ADC patients diagnosed and treated at the Heidelberg University Hospital between 2007 and 2020 (Table 1). ALK and EGFR status were determined at the Heidelberg Institute of Pathology using our routine diagnostic workflow of combined DNA and RNA sequencing starting from formalin-fixed and paraffin-embedded (FFPE) lung biopsies [22]. Tumors harboring activating EGFR mutations were classified as EGFR-positive, and tumors harboring oncogenic ALK fusions were classified as ALK-positive (Suppl. 1).

**Table 1:** Clinicopathological characteristics of the study cohort comprising 114 lung adenocarcinomas

Mutation subtype	ALK-positive	EGFR-positive	ALK/EGFR-negative
Number	31	40	43
Age: median (min.- max.)	58 (33–90)	69.5 (46–83)	64 (41–89)
Sex:			
Male	15 (48%)	7 (18%)	21 (49%)
Female	16 (52%)	33 (83%)	22 (51%)
Smoking history:			
Smoker	9 (29%)	15 (38%)	41 (95%)
Non-smoker	17 (55%)	25 (63%)	2 (5%)
Unknown	5 (16%)	0 (0%)	0 (0%)
Tumor stage:			
I	0 (0%)	0 (0%)	0 (0%)
II	1 (3%)	4 (10%)	0 (0%)
III	7 (23%)	10 (25%)	0 (0%)
IV	23 (74%)	26 (65%)	43 (100%)
Prior therapy:			
Naïve	31 (100%)	40 (100%)	26 (60%)
Chemotherapy	0 (0%)	0 (0%)	17 (40%)
Response to ICB	no ICB	no ICB	16 LTR^*^, 6 IR^†^, 21 RP^‡^

^*^Long-term responders

^†^Intermediate progressors

^‡^Rapid progressors

EGFR/ALK-positive patients were therapy-naïve, i.e., received neither TKI nor chemo- or immunotherapy prior to biopsy. ALK/EGFR-negative patients underwent biopsy immediately before start of ICB treatment and were further subdivided according to the subsequent ICB response in 16 long-term responders (LTR; durable response to ICB of 12 months or more), 21 rapid progressors (RP; disease progression within two months of ICB start) and 6 patients with an intermediate duration of response to ICB (IR). For all patients, only biopsies from the primary (lung) tumor with sufficient available mRNA for expression profiling were analyzed. The study was approved by the ethics committee of Heidelberg University (S-145/2017). The subcohort of ALK/EGFR-negative tumors was also analyzed in a study comparing the gene expression differences between LTR and RP [23].

### TCGA lung adenocarcinoma cohort

Gene expression and clinical data of the TCGA lung ADC cohort (LUAD) were downloaded from the PanCanAtlas webpage of the Genomic Data Commons repository (https://gdc.cancer.gov/about-data/publications/pancanatlas). Only samples diagnosed as lung adenocarcinoma and of the type “primary solid tumor” (code: 01) were included. Information on ALK fusion status was obtained from a gene fusion analysis based on RNA-Seq data [24]. The TCGA cohort included 5 ALK-positive, 57 EGFR-positive and 439 ALK/EGFR-negative lung ADC (Suppl. 2).

### Targeted gene expression profiling

Targeted mRNA expression profiling was conducted on the NanoString nCounter gene expression platform (NanoString Technologies, Seattle, WA) using a 770-gene panel (PanCancer Human IO 360 Panel) focused on genes connected with the interplay between tumor, tumor microenvironment and immune response in cancer. Per sample, 100 ng of total RNA in a final volume of 5 μl was mixed with a 3′ biotinylated capture probe and a 5′ reporter probe tagged with a fluorescent barcode sequence from the PanCancer IO 360 gene expression code set. Probes and target transcripts were hybridized at 65 °C for 18 h according to the manufacturer’s recommendations. Hybridized samples were run on the NanoString nCounter preparation station using the high-sensitivity protocol, in which excess capture and reporter probes are removed and transcript-specific complexes are immobilized on a streptavidin-coated cartridge. The samples were scanned at maximum resolution on the nCounter Digital Analyzer.

### Immunohistochemistry

For detection of FOXP3 and CD8 protein expression, 3 μm thick paraffin sections were prepared. Deparaffinization and tissue staining were performed using a Benchmark Ultra IHC Staining module according to standard protocols (FOXP3 Monoclonal Antibody, clone 236A/E7, eBioscience, Invitrogen, Thermo Fisher Scientific Inc., Waltham, MA and CONFIRM anti-CD8 Rabbit Monoclonal Primary Antibody, clone SP57, Roche, Mannheim, Germany). Staining was visualized using the Vectastain elite ABC detection system (Vector, Burlingame, CA, USA) and using 3,3′-Diaminobenzidine (Optiview DAB IHC Detection Kit or ultraView Universal DAB Detection Kit, Ventana, Roche, Mannheim, Germany) as chromogen. Hematoxylin was used for counterstaining of cell nuclei. Evaluation and scoring were performed by an expert pathologist using the image analysis software QuPath (Open Source Digital Pathology, https://github.com/qupath).

### Data analysis

Statistical analysis was performed using the programming language R. Sample normalization of the gene expression data was performed by fitting a linear model to negative and positive controls and subsequent housekeeping gene normalization. For the latter, the 20 panel genes with the lowest coefficient of variation and an expression level of at least 100 in the TCGA lung ADC dataset were used as housekeepers (*AKT1*, *API5*, *DNAJC14*, *EIF2B4*, *ELA*, *ERCC3*, *GLUD1*, *HDAC3*, *HMGB1*, *IFNAR1*, *MLH1*, *OAZ1*, *PUM1*, *RIPK1*, *SF3A1*, *STAT3*, *TBC1D10B*, *TLK2*, *TMUB2* and *UBB*). The gene expression profile of each sample was scaled by the median expression level of the housekeeping genes. Gene expression data were log2-transformed prior to statistical analysis and visualization.

The abundance of 14 immune cell populations (B cells, CD45+ cells, CD56dim NK cells, CD8+ T cells, cytotoxic cells, dendritic cells, exhausted CD8+ T cells, macrophages, mast cells, neutrophils, NK cells, T cells, Th1 cells and Treg cells) was estimated from the mRNA expression of marker genes as described and validated before [25]. The abundance of cell populations was reported on log2 scale. The “immunological distance” between two tumors was defined as the Manhattan distance between in the space of the 14 immune cell populations. Analyses were carried out on the following two levels: (1) on the level of the abundance of 14 immune cell populations and (2) on the level of the mRNA expression of 770 genes.

For heatmap displays, each of the cell populations (or genes) was centered (but not scaled) with respect to the mean abundance (or mean mRNA expression) over the samples. Hierarchical clustering was performed using Pearson correlations as similarity measure and the average linkage as measure of distances between clusters. Correlations between clusters and genetic subgroups were assessed using Fisher's exact test.

Differences between ALK-positive, EGFR-positive and ALK/EGFR-negative tumors were assessed for significance using the Kruskal–Wallis as omnibus test and the Wilcoxon test as post hoc test. The Benjamini–Hochberg procedure was used for p value correction, and lists of cell populations or genes were compiled controlling the false discovery rate (FDR) at 5%. KEGG Mapper was used to visualize the cytokine–cytokine receptor network (pathway hsa04060) [26].

## Results

The study cohort comprised 31 ALK-positive, 40 EGFR-positive and 43 ALK/EGFR-negative lung ADC patients (Table 1). Biopsies of each of the 114 primary tumors underwent gene expression profiling with an assay of 770 genes focused on immune-related genes. The abundance of 14 immune cell populations in the TME was estimated using an already established method [25].

### Overall level of immune cell infiltration

**Fig. 1:**
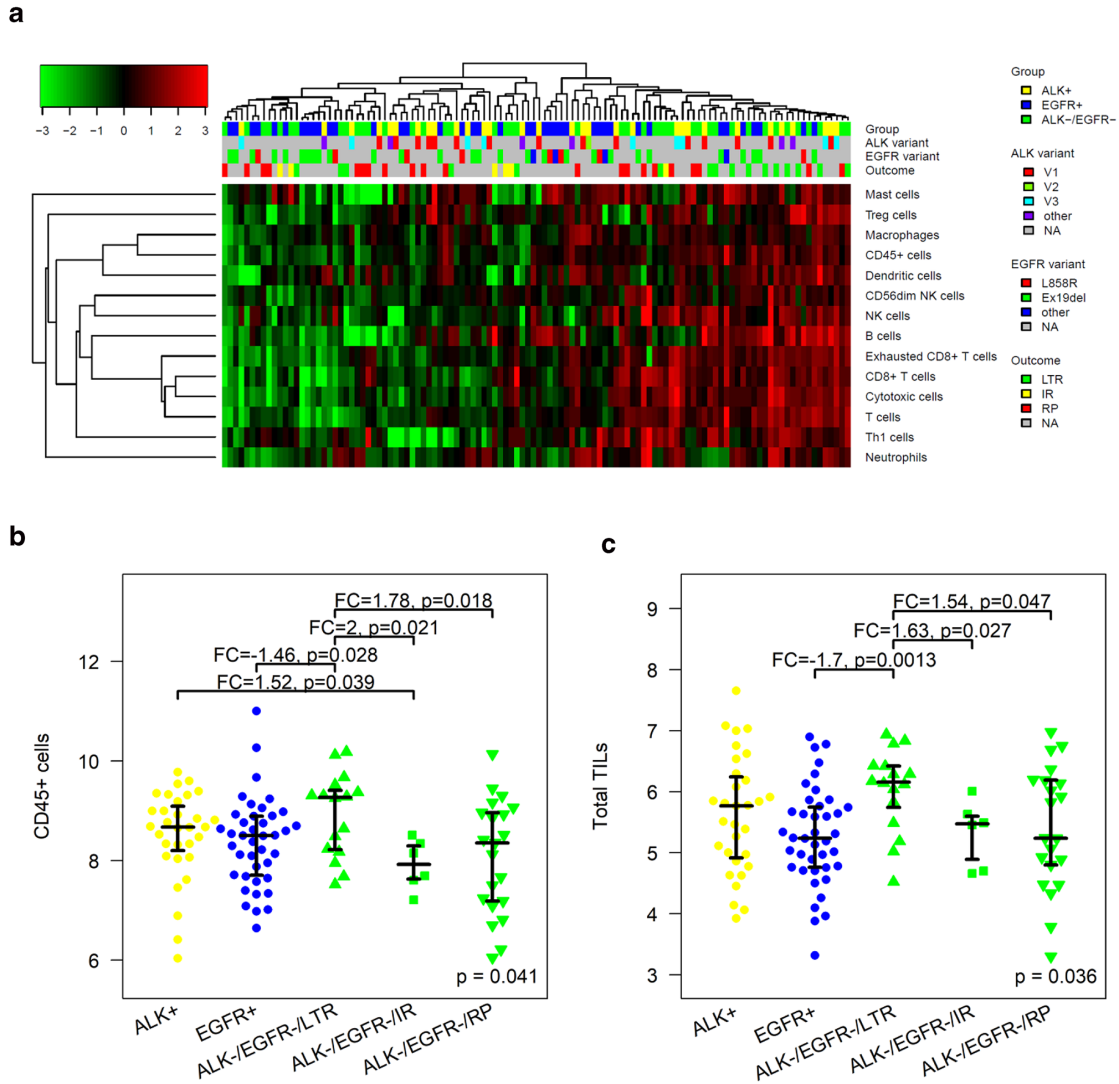
Immunological analysis of 114 lung adenocarcinomas by targeted gene expression profiling. **a** Clustering of the tumors by the abundance of 14 immune cell populations. **b** CD45+ cells were higher in EGFR/ALK-negative tumors that showed durable ICB response (long-term responders, LTR) compared to EGFR/ALK-negative tumors that progressed rapidly (rapid progressors, RP) and compared to EGFR-positive tumors. Levels of CD45+ cells between ALK-positive, EGFR-positive and rapid progressing ALK/EGFR-negative tumors did not differ significantly. IR = intermediate responders. **c** Similar as in B but for total TILs. Distributions are shown with median, lower and upper quartile

The levels of the immune cell populations were grouped by hierarchical clustering and visualized in a heatmap (Fig. 1a). The markers of cytotoxic cells, T cells, CD8+ T cells and exhausted CD8+ T cells clustered tightly together (all pairwise Spearman correlations *ρ* > 0.77). Moreover, macrophages and CD45+ cells showed a strong positive correlation (*ρ* = 0.67). The tumors clustered together in two main immunological groups, “cold” tumors (*n* = 58) and “hot” tumors (*n* = 56). The immunological groups did not correlate with the mutation type (ALK-positive, EGFR-positive or ALK/EGFR-negative) of the tumors (*p* = 0.82). Furthermore, the immunological groups did neither correlate with the type of ALK fusion (V1 vs. V3, *p* = 1) nor with the type of EGFR mutation (Ex19del vs. L858R, p = 0.69).

In line with the cluster analysis, neither the levels of CD45+ cells nor of total TILs (calculated as in [25]) correlated with the mutation type (*p* = 0.47 and *p* = 0.11). Additionally, we split the ALK/EGFR-negative tumors in long-term responders (LTR) and in rapid progressors (RP) with respect to ICB and carried out a four-group-analysis of ALK-positive tumors, EGFR-positive tumors, LTR and RP. Both, CD45+ cells and total TILs were significantly higher in LTR, but not in RP compared to EGFR-positive tumors (Fig. 1b, c). Neither CD45+ cells nor total TILs showed significant differences when comparing LTR or RP with ALK-positive tumors.

To test whether ALK/EGFR-positive tumors are “immunologically closer” to RP than to LTR, we operationalized the concept of immunological similarity by the introduction of an immunological distance based on the abundance of the 14 immune cell populations. It turned out that both ALK-positive tumors and EGFR-positive tumors were significantly closer to LTR than to RP tumors falsifying the hypothesis (Suppl. 3). This observation suggested distinct rather than common immunosuppressive features of the three subgroups, a hypothesis that we further addressed by comparative analyses of immune cell populations and gene expression profiles between the subgroups.

### Specific immune cell populations

**Fig. 2:**
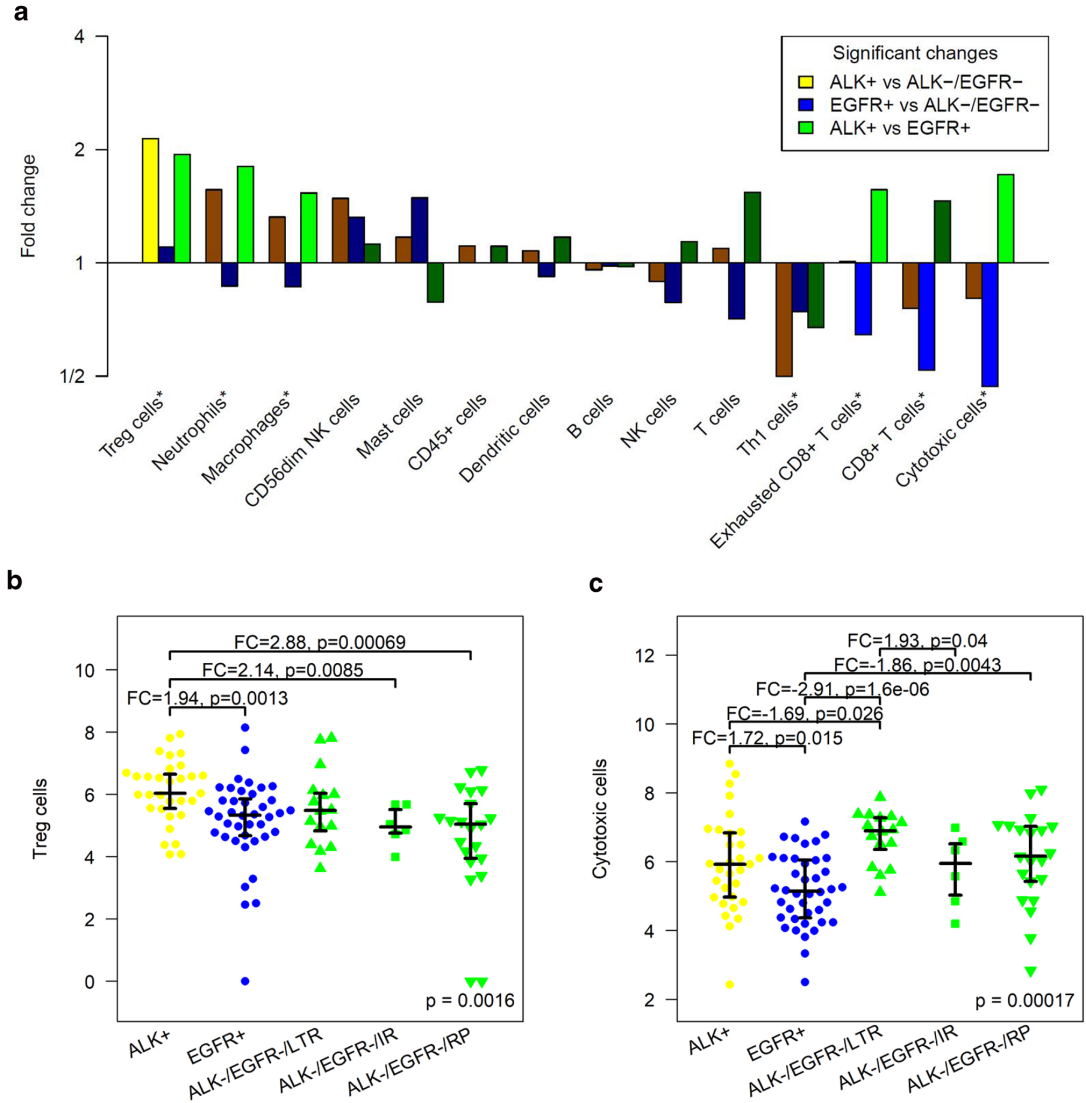
Levels of specific immune cell populations differ between ALK-positive, EGFR-positive and ALK/EGFR-negative lung adenocarcinomas. **a** Significantly higher Tregs in ALK-positive tumors compared to ALK/EGFR-negative tumors. Significantly lower cytotoxic cells, CD8+ T cells and exhausted CD8+ T cells in EGFR-positive tumors compared to ALK/EGFR-negative tumors. Significantly higher Tregs cells, neutrophils, macrophages, exhausted CD8+ T cells and cytotoxic cells in ALK-positive compared to EGFR-positive tumors. * = significant in omnibus test. **b** Significantly higher regulatory T cells in ALK-positive tumors compared to EGFR-positive tumors and to ALK/EGFR-negative tumors that progressed rapidly or intermediately after ICB. **c** Significantly lower cytotoxic cells in EGFR-positive tumors compared to ALK-positive tumors, to ALK/EGFR-negative tumors that showed durable ICB response and to ALK/EGFR-negative tumors that progressed rapidly under ICB

Seven of 14 immune cell populations were significantly different in ALK-positive, EGFR-positive and ALK/EGFR-negative tumors using omnibus testing (Fig. 2a, marked by*). Regulatory T cells (Tregs) were significantly higher in ALK-positive tumors compared to ALK/EGFR-negative tumors (FC = 2.1, *p* = 0.00047). Cytotoxic cells, CD8+ T cells and exhausted CD8+ T cells were significantly lower in EGFR-positive tumors compared to ALK/EGFR-negative tumors (FC =  − 2.1, *p* = 1.1E-05; FC =  − 1.9, *p* = 0.0037 and FC =  − 1.6, *p* = 0.0045). Here, ‘cytotoxic cells’ (markers genes: *PRF1*, *GZMA*, *GZMB*, *GZMH*, *GNLY*, *CTSW*, *KLRB1*, *KLRD1*, *KLRK1* and *NKG7*) refer to a broader cell population of granzyme releasing cells including cytotoxic T cell and cytotoxic NK cells compared to the more specific population of ‘CD8+ T cells’ (marker genes: *CD8A* and *CD8B*). Tregs, neutrophils, cytotoxic cells, exhausted CD8+ T cells and macrophages were significantly higher in ALK-positive compared to EGFR-positive tumors (FC = 1.9, *p* = 0.0013; FC = 1.8, *p* = 0.00078; FC = 1.7, *p* = 0.015; FC = 1.6, *p* = 0.017 and FC = 1.5, *p* = 0.00074). Thus, ALK-positive tumors stood out by significantly higher Tregs compared to EGFR-positive and ALK/EGFR-negative tumors, while EGFR-positive tumors stood out by significantly lower cytotoxic cells and exhausted CD8+ T cells compared to the two other subgroups.

In two more analyses, we investigated subgroups of the ALK/EGFR-negative tumors that were homogeneous regarding (1) prior treatment, (2) the duration of response to immune therapy, (3) smoking history and (4) TP53 mutation status. Firstly, the results obtained for the complete group of ALK/EGFR-negative tumors stayed correct for the subgroup of treatment-naïve tumors: Tregs were higher in ALK-positive cancer compared to treatment-naïve ALK/EGFR-negative cancer (FC = 2.3, *p* = 0.00015). Cytotoxic cells, CD8+ T cells and exhausted CD8+ T cells were lower in EGFR-positive cancer compared to treatment-naïve ALK/EGFR-negative cancer (FC =  − 2.2, p = 4.1E-05; FC =  − 2.0, *p* = 0.01 and FC =  − 1.7, *p* = 0.0082). Secondly, the group of ALK/EGFR-negative tumors was split in long-term responders (LTR) and rapid progressors (RP) under ICB (Fig. 2b, c, Suppl. 4). Tregs were significantly higher in ALK-positive tumors compared to RP (FC = 2.9, *p *= 0.00069), while a non-significant trend to higher levels (FC = 1.4, *p* = 0.094) was observed in comparison to LTR. Cytotoxic cells were significantly lower in EGFR-positive tumors compared to both RP and LTR (FC =  − 1.9, *p* = 0.0043 and FC =  − 2.9, *p* = 1.6E-06). Thirdly, levels of Tregs and cytotoxic cells did not differ significantly between smokers and non-smokers, while Tregs were enhanced in both ALK-positive tumors of smokers and non-smokers and cytotoxic cells were depleted in both EGFR-positive tumors of smokers and non-smokers (Suppl. 5A/B). Likewise, TP53 mutations did not confound the regulation patterns of Tregs and cytotoxic cells detected in the study cohort (Suppl. 5C/D).

Next, we investigated the validity of the findings in a cohort of earlier lung ADC (Suppl. 2). Of the three immune cell populations that were significantly reduced in EGFR-positive tumors compared to ALK/EGFR-negative tumors, the reduction of cytotoxic cells and CD8+ T was also found in the TCGA cohort (FC =  − 1.6, *p* = 0.015 and FC =  − 1.6, *p* = 0.0018). A significantly higher abundance of Tregs in ALK-positive tumors was not detected in the TCGA cohort, but this analysis was heavily underpowered with only five ALK-positive cases available.

### FOXP3 and CD8 protein expression

**Fig. 3:**
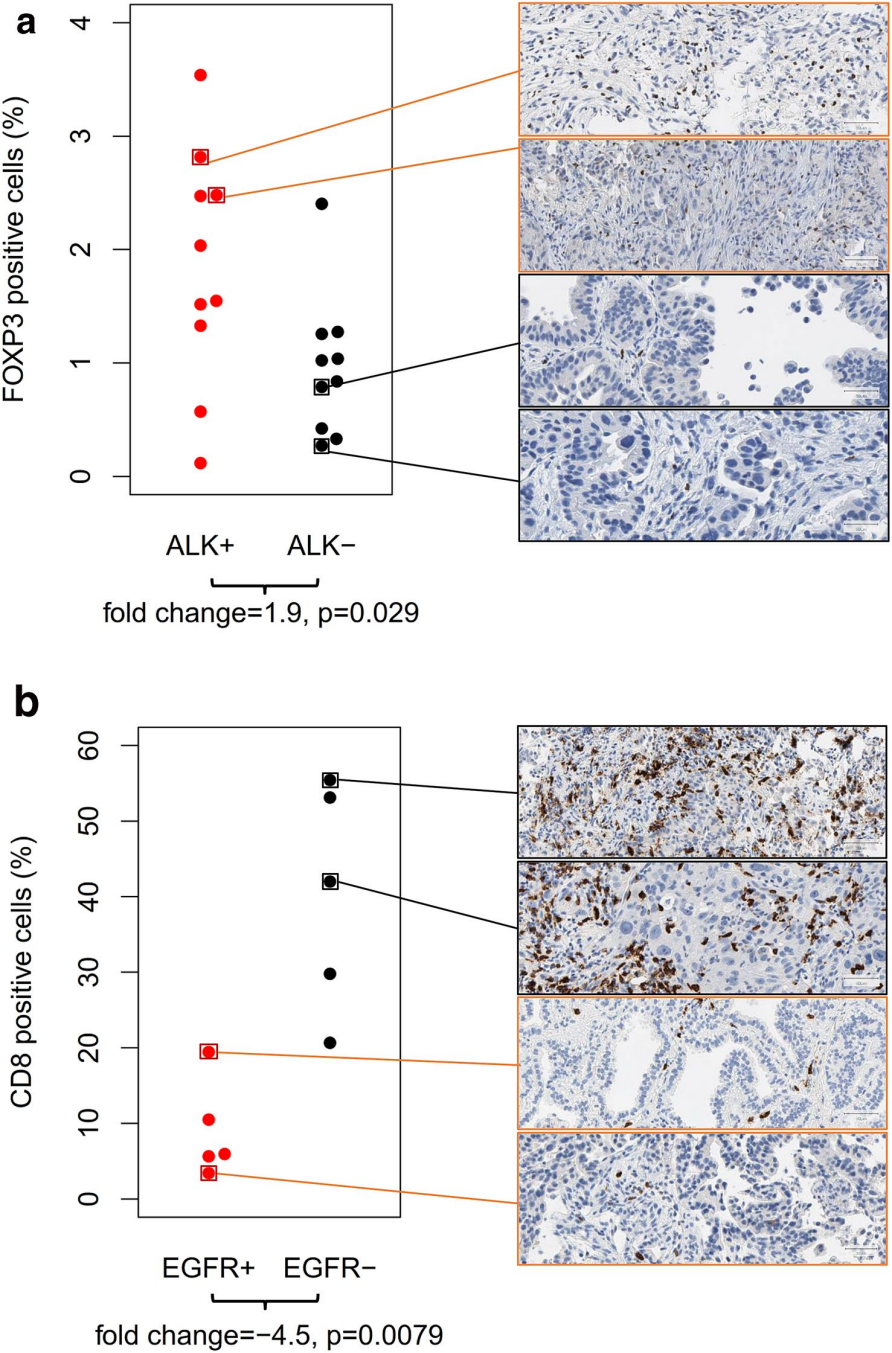
Immunohistochemical analysis of FOXP3 and CD8 protein expression in representative example cases of the study cohort. Positive cells were quantified as percentage of all (tumor and stroma) cells. **a** Comparison of ALK-positive tumors with high Treg mRNA marker and ALK-negative tumors with low Treg mRNA marker. The percentage of FOXP3-positive cells was significantly higher in the ALK-positive tumors (mean: 1.84%) than in ALK-negative tumors (mean: 0.96%). **b** Comparison of EGFR-positive tumors with low cytotoxic cell mRNA marker and EGFR-negative tumors with high cytotoxic cell mRNA marker. The percentage of CD8-positive cells was significantly lower in the EGFR-positive tumors (mean: 9%) compared to the EGFR-negative tumors (mean: 40%)

Representative example cases of the study cohort were analyzed using immunohistochemistry (Fig. 3). FOXP3 and CD8 protein expression were quantified by counting the positive cells in relation to all cells (tumor, immune and other cells) in the tissue section. The percentage of FOXP3-positive cells was lower than 4% in all of the twenty analyzed cases. We observed a diffuse distribution FOXP3-positive immune cells in the intra- and peritumoral stroma, while we did not observe any clusters of two or more adjacent FOXP3-positive cells. The percentage of FOXP3 was significantly higher (*p* = 0.029) in the ALK-positive cases (mean: 1.84%) compared to the ALK-negative cases (mean: 0.96%). The percentage of CD8-positive cells ranged between virtually 0% and 60% in the ten analyzed example cases. CD8-positive immune cells showed formation of small groups as well as of larger dense clusters. The percentage of CD8-positive cells was significantly lower (*p* = 0.0065) in the EGFR-positive cases (mean: 9%) compared to the EGFR-negative cases (mean: 40%). Altogether, the protein expression analysis confirmed enrichment of FOXP3-positive cells in ALK-positive cancer and depletion of CD8-postive cells in EGFR-positive cancer.

### PD-L1 expression

**Fig. 4:**
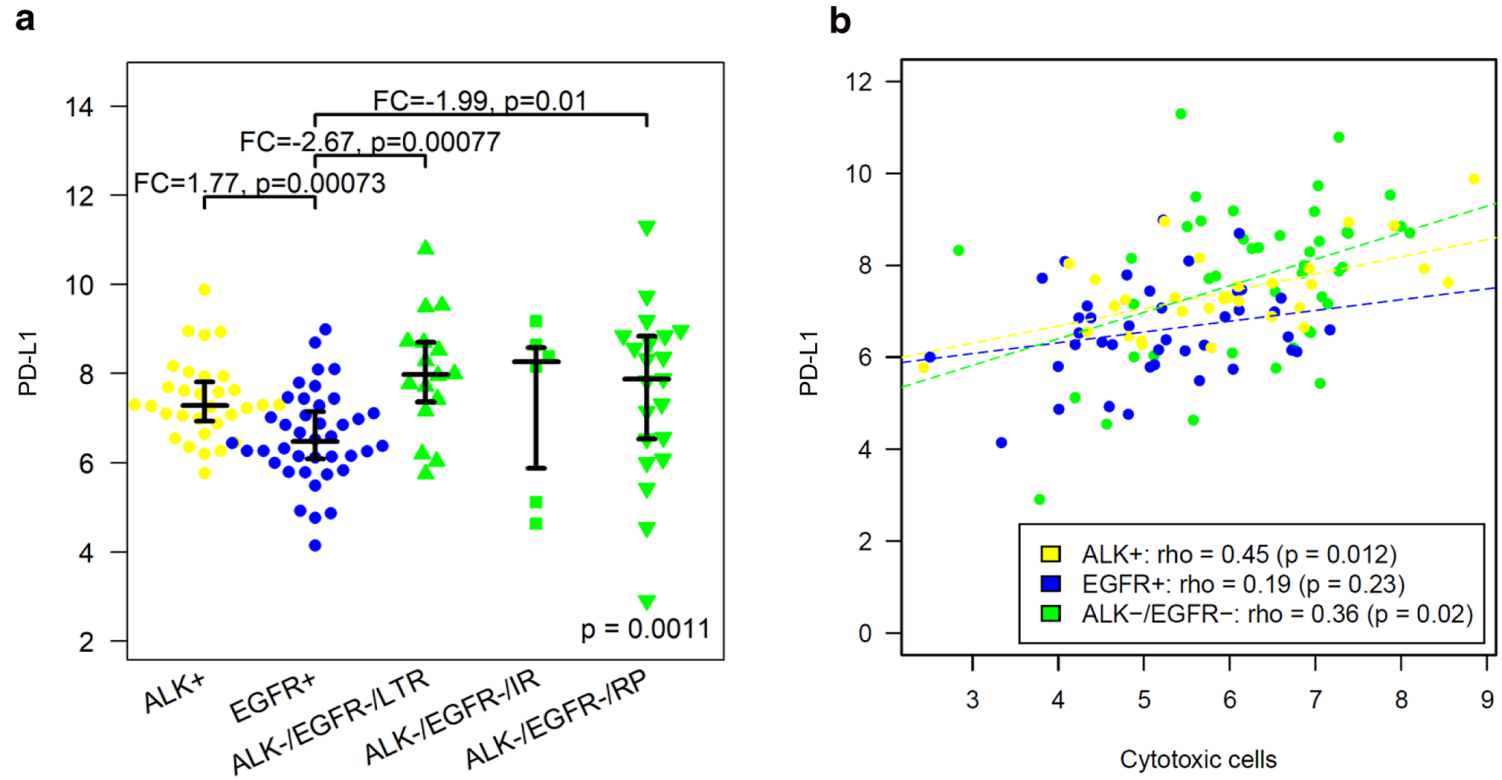
*PD-L1* expression in ALK-positive, EGFR-positive and ALK/EGFR-negative lung adenocarcinoma. **a** Significantly lower *PD-L1* mRNA expression in EGFR-positive tumors compared to ALK-positive tumors, ALK/EGFR-negative tumors that progressed rapidly after ICB and ALK/EGFR-negative tumors that responded durable to ICB. **b** *PD-L1* mRNA expression and levels of cytotoxic cells correlated positively significantly in ALK-positive and ALK/EGFR-negative tumors, while the correlation was weak and non-significant in EGFR-positive tumors

Levels of *PD-L1* mRNA were analyzed in a four-group-analysis (Fig. 4a). *PD-L1* mRNA expression was significantly lower in EGFR-positive tumors compared to the ALK-positive tumors (FC =  − 1.8, *p* = 0.00073) and compared to both groups of ALK/EGFR-negative tumors (LTR: FC =  − 2.7, p = 0.00077; RP: FC =  − 2.0, *p* = 0.01). Significant positive correlations with *PD-L1* mRNA expression were observed for eight of the 14 immune cell populations, namely for cytotoxic cells (*ρ* = 0.45), CD8+ T cells (*ρ* = 0.38), exhausted CD8+ cells (*ρ* = 0.34), neutrophils (*ρ* = 0.33), T cells (*ρ* = 0.32), Th1 cells (*ρ* = 0.31), dendritic cells (*ρ* = 0.3) and Treg cells (*ρ* = 0.28).

Thus, by far the strongest correlation was observed between *PD-L1* mRNA and cytotoxic cells (Fig. 4b). Partly, this correlation was induced by the incidence of low *PD-L1* expression and low abundance of cytotoxic cells in EGFR-positive tumors and at the same time higher levels of both markers in ALK-positive and ALK/EGFR-negative tumors. Additionally, *PD-L1* expression and cytotoxic cells correlated positively within some of the subgroups, namely in ALK-positive tumors (*ρ* = 0.45, *p* = 0.012) and in ALK/EGFR-negative tumors (*ρ* = 0.36, *p* = 0.02), but not EGFR-positive tumors where the correlation was not significant.

### Gene expression analysis

In omnibus testing, 289 of 770 investigated genes showed significantly different expression levels in ALK-positive, EGFR-positive and ALK/EGFR-negative tumors (FDR = 5%, Suppl. 6). In detail, 145 genes were differentially expressed between ALK-positive and ALK/EGFR-negative tumors, 122 genes were differentially expressed between EGFR-positive and ALK/EGFR-negative tumors, and 192 genes were differentially expressed between ALK-positive and EGFR-positive tumors. The results could be partially confirmed by analyzing the independent TCGA cohort of early stage lung ADC with a match of 44 genes (30%), 63 genes (52%) and 28 genes (15%) of the respective differentially expressed genes. Confirmation rates were imperfect, but clearly above the random baseline of 5% corresponding to the *p* value threshold 0.05. A bias was expected, because of the limited sample size (only five tumors with ALK fusions in the TCGA cohort), clinical differences between the in-house and the TCGA cohort (later stage vs. earlier stage tumors) and different analysis platforms (NanoString nCounter vs. RNA-Seq).

**Fig. 5:**
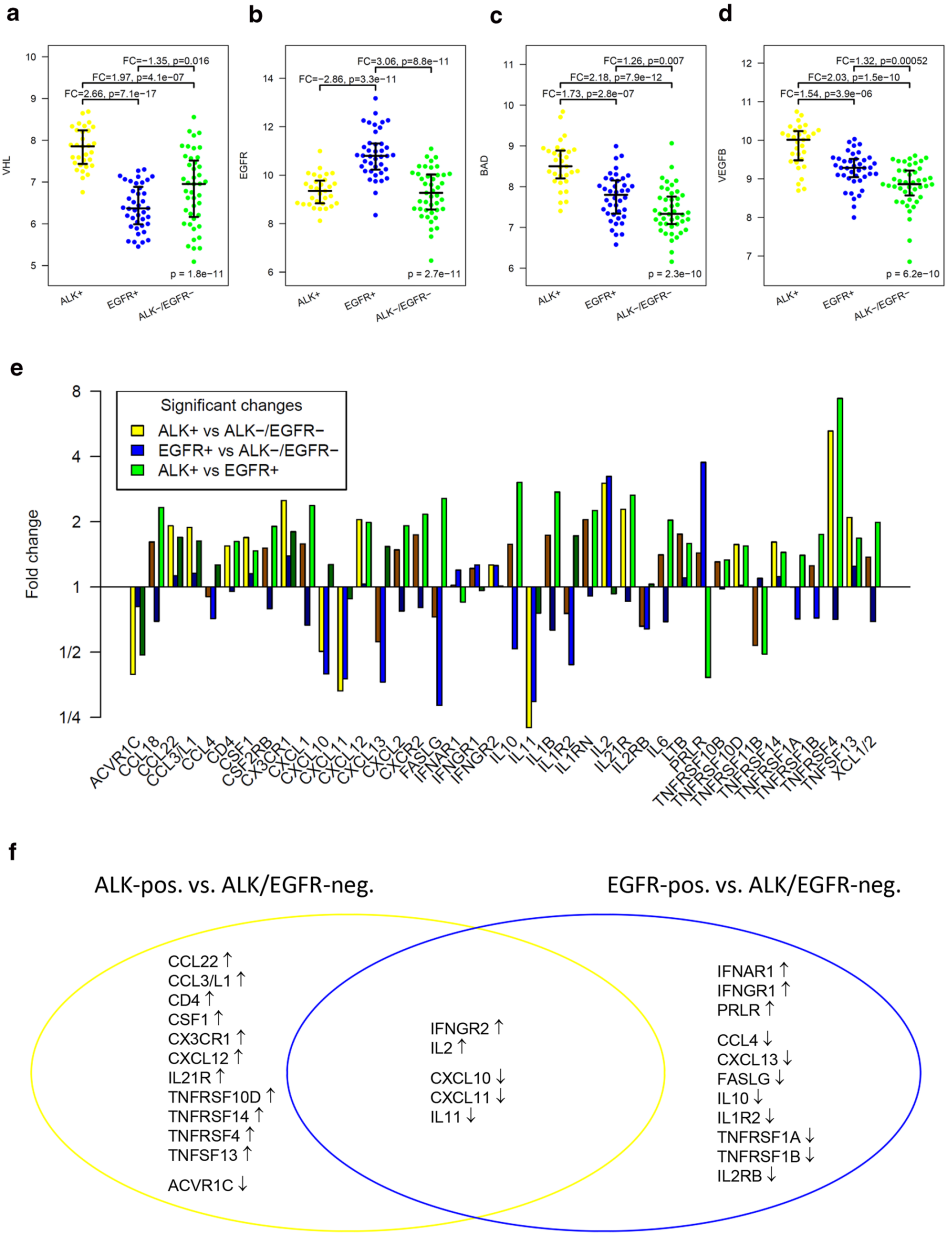
Differential gene expression between ALK-positive, EGFR-positive and ALK/EGFR-negative lung adenocarcinoma**. a**–**d** Expression levels of most significantly differentially expressed genes in omnibus testing: **a** *VHL* was overexpressed in ALK-positive tumors compared to both EGFR-positive tumors and ALK/EGFR-negative tumors. **b** *EGFR* was overexpressed in EGFR-positive tumors compared to both ALK-positive tumors and ALK/EGFR-negative tumors.** c** *BAD* showed the highest expression in ALK-positive tumors, an intermediate expression in EGFR-positive tumors and the lowest expression in ALK/EGFR-negative tumors.** d** *VEGFB* showed the same decreasing expression pattern as BAD. **e**–**f** Analysis of the cytokine–cytokine receptor system: **e** Fold changes of the 40 differentially expressed of the 121 investigated cytokines and cytokine receptors. **f** Twelve genes were differentially expressed exclusively between ALK-positive and ALK/EGFR-negative tumors, eleven genes were differentially expressed exclusively between EGFR-positive and ALK/EGFR-negative tumors, while five genes were differentially expressed in both comparisons. ↑ = upregulation, ↓ = downregulation

These were the four most significant gene expression changes detected by the omnibus test in the study cohort (Fig. 5a–d): *VHL* was overexpressed in ALK-positive tumors compared to both EGFR-positive tumors and ALK/EGFR-negative tumors. *EGFR* was overexpressed in EGFR-positive tumors compared to both ALK-positive tumors and ALK/EGFR-negative tumors. *BAD* showed the highest expression in ALK-positive tumors, an intermediate expression in EGFR-positive tumors and the lowest expression in ALK/EGFR-negative tumors. *VEGFB* showed the same decreasing expression pattern as *BAD*. The overexpression of *EGFR* in EGFR-positive tumors compared to ALK-positive and ALK/EGFR-negative tumors was also found in the TCGA cohort (FC = 2.2, *p* = 0.0097 and FC = 3.1, *p* = 3.1E-16), while we did not detect differential expression of the other three genes.

### Analysis of the cytokine–cytokine receptor signaling network

Beyond the estimation of immune cell abundance in the TME, gene expression profiling offers the opportunity to gain insight into the regulation of immune response. Of 294 genes annotated in the KEGG map of cytokines and cytokine receptors, 121 genes (41%) were covered by the used targeted expression assay. Of these, 40 genes (33%) were differentially expressed, 17 between ALK-positive and ALK/EGFR-negative tumors, 16 between EGFR-positive and ALK/EGFR-negative tumors and 26 between ALK-positive and EGFR-positive tumors (Fig. 5e). Compared to ALK/EGFR-negative tumors, five genes were differently expressed in both ALK-positive and EGFR-positive tumors, while twelve genes showed differential expression solely in ALK-positive tumors and eleven genes showed differential expression solely in EGFR-positive tumors (Fig. 5f). We assigned the changes to the map of the interaction of cytokines and receptors (Suppl. 7).

*IL2*, an interferon that is important for the proliferation of T and B lymphocytes and in particular for the differentiation of CD4+ T cells to T helper cells and Tregs, was overexpressed in ALK-positive and in EGFR-positive tumors compared to ALK/EGFR-negative tumors (FC = 3.0, *p* = 0.00013 and FC = 3.2, *p* = 3.0E-05). *IL11*, an interferon that is known to stimulate the T cell-dependent development of immunoglobulin-producing B cells, was underexpressed in ALK-positive and in EGFR-positive tumors compared to ALK/EGFR-negative tumors (FC =  − 4.5, *p* = 2.7E-05 and FC =  − 3.4, *p* = 0.00024). Tumor necrosis factor *TNFRSF4*, suggested to play a role in CD4+ T cell response as well as in T cell-dependent B cell proliferation and differentiation, was strongly overexpressed in ALK-positive tumors compared to ALK/EGFR-negative tumors and compared to EGFR-positive tumors (FC = 5.2, *p* = 4.0E-07 and FC = 7.4, *p* = 1.0E-07). *FASLG*, a cytokine that binds to the receptor FAS that transduces apoptotic signals in cells, was underexpressed in EGFR-positive tumors compared to ALK/EGFR-negative tumors and compared to ALK-positive tumors (FC =  − 3.5, *p* = 2.5E-05 and FC =  − 2.6, *p* = 0.0033), an observation possibly related to the lower abundance of cytotoxic cells in EGFR-positive tumors. Prolactin receptor (*PRLR*), which has been suggested as a therapeutic target in subgroups of breast and of prostate cancer [27], was overexpressed in EGFR-positive tumors compared to ALK/EGFR-negative tumors and compared to ALK-positive tumors (FC = 3.8, *p* = 0.0004 and FC = 2.6, *p* = 0.013).

## Discussion

About 45% to 50% of all NSCLC show a variety of mutually exclusive genetic driver lesions that can be therapeutically exploited. Collectively, EGFR mutations and ALK fusions represent the largest subgroup comprising approx. 20–25%. While these tumors respond well to TKIs, the efficacy of ICB is generally poor despite tremendous trial efforts in the last few years. These observations led to the hypothesis of a poorly immunogenic “immune desert” that prevents successful application of ICB. Early studies of various sample sizes and disease stages investigating immune cell compositions of NSCLC by either immunohistochemistry or flow cytometry revealed the presence of a broad range of infiltrating immune cells including T cells, NK cells, macrophages, B cells, dendritic cells and granulocytes [28–35].

Complementing and extending these studies, we compiled a retrospective cohort of carefully clinically annotated and confirmed ALK- and EGFR-positive NSCLC, molecularly analyzed at the Heidelberg routine diagnostic laboratory [22]. We profiled routine diagnostic formalin-fixed and paraffin-embedded biopsies after mutational analysis by NGS using the NanoString nCounter technology [36]. This technology, which can be directly applied in a routine diagnostic setting and is tissue sparing, facilitates focused analysis of mRNA profiles of 770 genes with fragmented nucleic acids as input material. It enables a multilayered analysis of specific immune cell types as well as expression levels of cytokines, cytokine receptors and other immune genes.

In the first analysis presented here, we identified two groups of immunologically “cold” and “hot” tumors, which were not restricted to a specific subgroup (ALK-positive, EGFR-positive or ALK/EGFR-negative) or oncogene variant (e.g., ALK V1 vs. V3 or EGFR Ex19del vs. L858R). In the analysis of specific cell populations, different modifications of the TME were detected in ALK-positive and in EGFR-positive tumors compared to ALK/EGFR-negative tumors, respectively: While ALK-positive cases displayed a Treg enriched TME, EGFR-positive cases showed a cytotoxic cell-depleted TME suggesting different mechanisms that abrogate a strong and sustained immune cell response. These two different TMEs appear to be governed by specific immunomodulatory networks, which might be clinically exploitable [37]. Our results confirm other studies analyzing genetic subtypes of NSCLC that found low infiltrates of T cells and CD8+ cells in EGFR-mutant tumors [17, 18]. By contrast, data for ALK-positive NSCLC are highly limited, a few groups identified low CD8 populations in these tumors as well [21, 38]. A more recent study presented reported changes in the immune TME associated with TKI inhibition [39].

For ALK-positive disease, we observed a significant downregulation of *CXCL10* and *CXCL11* negatively influencing general T cell recruitment [40], while at the same time upregulating *CXCL12* [41–43] as well as *CCL22* [44–46] which attract CXCR4+ Tregs. Consistent with the suggested regulation mechanism, T cell levels in the study cohort correlated significantly with *CXCL10* and *CXCL11* (*ρ* = 0.44, *p* = 1.6E-06 and *ρ* = 0.47, *p* = 1.1E-07). Tregs correlated significantly with *CCL22* (*ρ* = 0.64, *p* = 2.8E-14), while the correlation of Tregs and *CXCL12* was not significant. Downregulation of *CXCL10* and *CXCL11* was also observed in EGFR-positive disease.

Comparing ALK-positive and EGFR-positive disease, we noted strong upregulation of *IL10* in ALK-positive tumors. *IL10* is involved in negative feedback mechanisms which decrease the antigen-presenting activity of dendritic cells and inhibit cytotoxic functions and cytokine-release of T and NK lymphocytes [47]. Similarly, pleiotropic *IL6* was upregulated, which was reported to negatively influence T cell response and innate immunity [48, 49]. Notably, the upregulation of *CSF1* and *CCL18*—as identified in the TME of ALK-positive tumors —increases type 2 (M2) tumor-associated macrophages (TAM) which have tumorigenic functions and are known to contribute to immune evasion [50, 51]. *VEGFB* was also upregulated in ALK-positive NSCLC. This might be explained by angiogenic effects but could also be due to potential immunosuppressive effects recently described for VEGF signaling [52, 53]. Interestingly, we identified upregulation of some costimulatory molecules influencing T cell response, including *TNFRSF4* (aka *OX40* [54]). We also observed upregulation of *TNFSF13* (aka *APRIL*), a molecule influencing B cell development. The latter two phenomena may represent an unfruitful attempt to overcome a generally immune response deprived TME.

The proinflammatory cytokine *IL2* was found to be upregulated in both ALK- and EGFR-positive tumors, while *IL11*, a member of the *IL6* family, was found to be downregulated in both subgroups. *IL2* is known to modulate the development and expansion of Tregs exerting immunosuppressive effects [55]. High *IL11* has been linked to cell proliferation and tumorigenesis in in vitro and in vivo models of NSCLC as well as to unfavorable prognosis [56].

EGFR-positive tumors feature a CD8+ deprived environment that is modulated by downregulation of CCL4, a molecule that supports recruitment of CD103+ dendritic cells which active CD8+ cells [57] as well as downregulated CXCL10, the ligand for CXCR3 on cytotoxic T cells and downregulated CXCL11 negatively modulating CD8+ T cell migration [58, 59]. *IFNGR1* and *IFNGR2* were upregulated, possibly as a compensatory yet non-successful mechanism to overcome impaired CD8+ T cell-mediated immune response.

At the Heidelberg Institute of Pathology, mRNA extracts of lung cancer biopsies are acquired within the routine workflow of lung cancer biopsies and used for gene fusion analysis [22]. For most of the tumors in the study cohort, leftovers of such extract were available and suitable for gene expression profiling. Thus, targeted gene expression represents a tissue-saving method to read out multi-dimensional information on the immune TME from diagnostic lung cancer biopsies.

It is a limitation of gene expression analysis of bulk tissues that expression levels are summarized over all cells in the tissue, while most of the human genes are expressed in various cell types, e.g., in cancer cells and in different other cells in the TME. Thus, in general, the cells types that are causative for the detected gene expression changes cannot be identified from a bulk tissue analysis. Nevertheless, there are transcripts that are expressed by a only a single cell type and conversely there are cell types that can be characterized by the expression of specific marker genes, a methodology that has been exploited to distinguish between 14 immune cell populations in current study.

An advantage of nucleic acid-based technologies is the parallel monitoring of a high number of genes, while spatial resolved technologies such as immunohistochemistry and multispectral imaging usually allow the monitoring of only one or a few genes in parallel and are more difficult to generalize. Thus, nucleic acid-based technologies can be very helpful for screening and knowledge discovery, while spatial resolved technologies should be employed for validation and in-depth cell type-specific analysis. In the current study, we validated an accumulation of Tregs in ALK-positive tumors and a depletion of CD8+ T cells in EGFR-positive tumors using IHC.

Spatial tumor heterogeneity represents an additional potential limitation for studies in metastatic NSCLC based on a single biopsy of each patient, but this method of sample collection is currently used in the routine molecular pathology.

Collectively, our data revealed two different types of TME modification in ALK- and EGFR-positive NSCLC, respectively, which we term Treg-modulated and CD8-modulated immune TME, each accompanied by a specific characteristic of cytokine signaling. Therefore, tailored therapy strategies will probably be required for oncogene-addicted tumors and we expect detailed profiling of the TME to be instrumental for guiding the selection of appropriate immunomodulatory partners to ICB. Furthermore, our results support the need for exploration of new therapeutic approaches to transform these TME into “hot” states [60]. This may be achievable by opportunistic combinations of ICB with radio- and chemotherapy, or by more subtle, specific approaches that either inhibit specific immunosuppressive agents enriched in “cold” tumors or supplement and boost proinflammatory molecules depleted in “cold” tumors. The detection of significant immune reactivity in oncogene-addicted NSCLC, in contrast the conception of an absolute “immune desert,” suggests that well-aimed use of immunomodulators currently explored in various pre-clinical studies and clinical trials [37, 61] may also hold promise in EGFR- and ALK-positive NSCLC.

### Supplementary Information

Below is the link to the electronic supplementary material.

Supplementary file1 (PDF 424 KB)

Supplementary file2 (PDF 95 KB)

Supplementary file3 (PDF 259 KB)

Supplementary file4 (PDF 158 KB)

Supplementary file5 (PDF 422 KB)

Supplementary file6 (PDF 682 KB)

Supplementary file7 (PDF 380 KB)

## Abbreviations

ADC: Adenocarcinoma
ALK-positive tumor: Tumor with activating ALK gene fusion
ALK/EGFR-negative tumor: Tumor that is neither ALK-positive nor EGFR-positive
EGFR-positive tumor: Tumor with activating somatic mutation in EGFR
FC: Fold change
ICB: Immune checkpoint blockade
LTR: Long-term responders (at least one year after start of ICB)
NSCLC: Non-small cell lung cancer
RP: Rapid progressors (within two months after start of ICB)
rho: Spearman correlation coefficient
TILs: Tumor-infiltrating lymphocytes
TKI: Tyrosine kinase inhibitor
TMB: Tumor mutational burden
TME: Tumor microenvironment
Tregs: Regulatory T cells
