# Supplementary material for: Deciphering the immunosuppressive tumor microenvironment in ALK- and EGFR-positive lung adenocarcinoma
Source: Cancer Immunol Immunother. 2021 Jun 14;71(2):251–65. doi: 10.1007/s00262-021-02981-w (PMC8783861; doi:10.1007/s00262-021-02981-w)
Supplement: Supplementary file 1 — Supplementary file1 (PDF 424 KB) [file 262_2021_2981_MOESM1_ESM.pdf]

Supplement 1: Study cohort of 114 lung adenocarcinoma patients

id	age	sex	subgroup	EGFR mutation	EGFR type	ALK mutation	ALK type	TP53 mutation	tumor stage	prior therapy	response to ICB	smoking
LUAD6	55	female	ALK-/EGFR-	normal	normal	normal	normal	deleterious	IV	chemotherapy	RP	smoker
LUAD7	59	female	ALK-/EGFR-	normal	normal	normal	normal	deleterious	IV	chemotherapy	LTR	ex-smoker
LUAD11	51	female	ALK-/EGFR-	normal	normal	normal	normal	deleterious	IV	chemotherapy	RP	ex-smoker
LUAD12	73	male	ALK-/EGFR-	normal	normal	normal	normal	normal	IV	chemotherapy	LTR	smoker
LUAD17	65	male	ALK-/EGFR-	normal	normal	normal	normal	normal	IV	naive	IR	smoker
LUAD18	63	female	ALK-/EGFR-	normal	normal	normal	normal	deleterious	IV	naive	LTR	smoker
LUAD21	47	male	ALK-/EGFR-	normal	normal	normal	normal	normal	IV	chemotherapy	IR	smoker
LUAD22	81	male	ALK-/EGFR-	normal	normal	normal	normal	normal	IV	naive	LTR	ex-smoker
LUAD23	41	female	ALK-/EGFR-	normal	normal	normal	normal	deleterious	IV	chemotherapy	RP	smoker
LUAD32	59	female	ALK-/EGFR-	normal	normal	normal	normal	normal	IV	naive	IR	ex-smoker
LUAD33	65	male	ALK-/EGFR-	normal	normal	normal	normal	deleterious	IV	chemotherapy	LTR	smoker
LUAD34	68	male	ALK-/EGFR-	normal	normal	normal	normal	normal	IV	chemotherapy	LTR	smoker
LUAD35	72	female	ALK-/EGFR-	normal	normal	normal	normal	deleterious	IV	chemotherapy	RP	ex-smoker
LUAD37	56	female	ALK-/EGFR-	normal	normal	normal	normal	normal	IV	naive	RP	smoker
LUAD38	67	female	ALK-/EGFR-	normal	normal	normal	normal	deleterious	IV	naive	RP	ex-smoker
LUAD52	73	female	ALK-/EGFR-	normal	normal	normal	normal	normal	IV	naive	LTR	smoker
LUAD56	49	male	ALK-/EGFR-	normal	normal	normal	normal	deleterious	IV	chemotherapy	RP	ex-smoker
LUAD57	65	female	ALK-/EGFR-	normal	normal	normal	normal	deleterious	IV	naive	RP	ex-smoker
LUAD58	54	male	ALK-/EGFR-	normal	normal	normal	normal	deleterious	IV	naive	RP	smoker
LUAD59	70	male	ALK-/EGFR-	normal	normal	normal	normal	normal	IV	naive	RP	ex-smoker
LUAD61	61	male	ALK-/EGFR-	normal	normal	normal	normal	normal	IV	chemotherapy	RP	ex-smoker
LUAD63	46	female	ALK-/EGFR-	normal	normal	normal	normal	normal	IV	chemotherapy	IR	ex-smoker
LUAD64	57	female	ALK-/EGFR-	normal	normal	normal	normal	deleterious	IV	chemotherapy	RP	ex-smoker
LUAD65	65	male	ALK-/EGFR-	normal	normal	normal	normal	deleterious	IV	naive	RP	ex-smoker
LUAD66	82	female	ALK-/EGFR-	normal	normal	normal	normal	normal	IV	naive	RP	non-smoker
LUAD67	50	female	ALK-/EGFR-	normal	normal	normal	normal	normal	IV	chemotherapy	RP	ex-smoker
LUAD69	57	female	ALK-/EGFR-	normal	normal	normal	normal	normal	IV	chemotherapy	IR	smoker
LUAD70	70	female	ALK-/EGFR-	normal	normal	normal	normal	deleterious	IV	naive	IR	ex-smoker
LUAD72	56	female	ALK-/EGFR-	normal	normal	normal	normal	deleterious	IV	naive	RP	ex-smoker
LUAD74	72	male	ALK-/EGFR-	normal	normal	normal	normal	deleterious	IV	naive	LTR	ex-smoker
LUAD75	72	male	ALK-/EGFR-	normal	normal	normal	normal	normal	IV	naive	LTR	smoker
LUAD76	89	male	ALK-/EGFR-	normal	normal	normal	normal	normal	IV	naive	LTR	non-smoker
LUAD77	64	male	ALK-/EGFR-	normal	normal	normal	normal	normal	IV	chemotherapy	LTR	smoker
LUAD78	79	male	ALK-/EGFR-	normal	normal	normal	normal	normal	IV	naive	LTR	ex-smoker
LUAD79	59	male	ALK-/EGFR-	normal	normal	normal	normal	deleterious	IV	naive	LTR	ex-smoker
LUAD80	66	female	ALK-/EGFR-	normal	normal	normal	normal	deleterious	IV	naive	LTR	smoker
LUAD81	72	male	ALK-/EGFR-	normal	normal	normal	normal	normal	IV	naive	RP	ex-smoker
LUAD82	62	male	ALK-/EGFR-	normal	normal	normal	normal	unknown	IV	chemotherapy	LTR	smoker

LUAD83	68	male	ALK-/EGFR-	normal	normal	normal	normal	normal	IV	naive	RP	smoker
LUAD84	65	female	ALK-/EGFR-	normal	normal	normal	normal	normal	IV	naive	RP	smoker
LUAD85	63	male	ALK-/EGFR-	normal	normal	normal	normal	deleterious	IV	naive	LTR	smoker
LUAD88	58	female	ALK-/EGFR-	normal	normal	normal	normal	normal	IV	naive	RP	ex-smoker
LUAD89	53	female	ALK-/EGFR-	normal	normal	normal	normal	normal	IV	naive	RP	ex-smoker
EGFR1	52	male	EGFR+	p.Leu747_Ala750delinsPro	Ex19del	normal	normal	deleterious	IV	naive	no IBC	non-smoker
EGFR2	76	female	EGFR+	p.Glu746_Ala750del	Ex19del	normal	normal	deleterious	II	naive	no IBC	ex-smoker
EGFR3	81	male	EGFR+	p.Leu858Arg	L858R	normal	normal	normal	III	naive	no IBC	non-smoker
EGFR5	65	female	EGFR+	p.Glu746_Ala750del	Ex19del	normal	normal	normal	III	naive	no IBC	non-smoker
EGFR6	81	female	EGFR+	p.Leu747_Ser752del	Ex19del	normal	normal	normal	IV	naive	no IBC	non-smoker
EGFR7	58	female	EGFR+	p.Glu746_Ala750del	Ex19del	normal	normal	normal	IV	naive	no IBC	non-smoker
EGFR11	79	female	EGFR+	p.Leu858Arg	L858R	normal	normal	deleterious	IV	naive	no IBC	non-smoker
EGFR12	70	female	EGFR+	p.Ser752_Ile759del	Ex19del	normal	normal	normal	III	naive	no IBC	non-smoker
EGFR15	78	female	EGFR+	p.Leu747_Thr751delinsPro	Ex19del	normal	normal	deleterious	III	naive	no IBC	non-smoker
EGFR17	53	female	EGFR+	p.Leu747_Ser752del	Ex19del	normal	normal	deleterious	IV	naive	no IBC	smoker
EGFR18	58	female	EGFR+	p.Leu747_Pro753delinsSer	Ex19del	normal	normal	deleterious	II	naive	no IBC	ex-smoker
EGFR21	75	female	EGFR+	p.Glu746_Ala750del	Ex19del	normal	normal	normal	IV	naive	no IBC	non-smoker
EGFR22	77	female	EGFR+	p.Glu746_Ala750del	Ex19del	normal	normal	deleterious	III	naive	no IBC	non-smoker
EGFR25	70	female	EGFR+	p.Leu858Arg	L858R	normal	normal	normal	III	naive	no IBC	ex-smoker
EGFR26	67	female	EGFR+	p.Leu858Arg	L858R	normal	normal	unknown	II	naive	no IBC	ex-smoker
EGFR27	56	male	EGFR+	p.Glu746_Ala750del	Ex19del	normal	normal	normal	IV	naive	no IBC	non-smoker
EGFR32	57	female	EGFR+	p.Glu746_Ala750del	Ex19del	normal	normal	deleterious	III	naive	no IBC	non-smoker
EGFR33	70	female	EGFR+	p.Glu746_Ala750del	Ex19del	normal	normal	normal	IV	naive	no IBC	ex-smoker
EGFR35	78	male	EGFR+	p.Leu858Arg	L858R	normal	normal	deleterious	IV	naive	no IBC	smoker
EGFR36	54	female	EGFR+	p.Glu746_Ala750del	Ex19del	normal	normal	normal	IV	naive	no IBC	non-smoker
EGFR37	74	female	EGFR+	p.Glu746_Ala750del	Ex19del	normal	normal	deleterious	IV	naive	no IBC	non-smoker
EGFR39	72	female	EGFR+	p.Glu746_Ser752delinsVal	Ex19del	normal	normal	normal	IV	naive	no IBC	non-smoker
EGFR44	77	female	EGFR+	p.Leu747_Ala750delinsPro	Ex19del	normal	normal	normal	IV	naive	no IBC	non-smoker
EGFR46	82	female	EGFR+	p.Leu861Gln	other	normal	normal	deleterious	IV	naive	no IBC	ex-smoker
EGFR49	46	female	EGFR+	p.Leu833Val; p.His835Leu	other	normal	normal	deleterious	III	naive	no IBC	smoker
EGFR50	63	female	EGFR+	p.Leu858Arg	L858R	normal	normal	deleterious	III	naive	no IBC	ex-smoker
EGFR51	51	female	EGFR+	p.Leu858Arg	L858R	normal	normal	deleterious	IV	naive	no IBC	non-smoker
EGFR53	69	female	EGFR+	p.Leu747_Ala750del	Ex19del	normal	normal	normal	III	naive	no IBC	non-smoker
EGFR56	83	female	EGFR+	p.Glu746_Ala750del	Ex19del	normal	normal	normal	IV	naive	no IBC	non-smoker
EGFR58	60	female	EGFR+	p.Glu746_Ser752delinsVal	Ex19del	normal	normal	deleterious	II	naive	no IBC	smoker
EGFR60	58	male	EGFR+	p.Glu746_Ala750del	Ex19del	normal	normal	normal	IV	naive	no IBC	non-smoker
EGFR61	82	female	EGFR+	p.Glu709Lys; p.Gly719Ala	other	normal	normal	normal	IV	naive	no IBC	non-smoker
EGFR66	65	female	EGFR+	p.Leu747_Ser752delinsGln	Ex19del	normal	normal	deleterious	IV	naive	no IBC	ex-smoker
EGFR68	52	female	EGFR+	p.Leu858Arg	L858R	normal	normal	unknown	IV	naive	no IBC	smoker
EGFR69	53	female	EGFR+	p.Glu746_Ala750del	Ex19del	normal	normal	deleterious	IV	naive	no IBC	non-smoker
EGFR70	52	female	EGFR+	p.Glu709_Thr710delinsAsp	other	normal	normal	deleterious	IV	naive	no IBC	non-smoker

EGFR72	59	female	EGFR+	p.Glu746_Ala750del	Ex19del	normal	normal	deleterious	IV	naive	no IBC	smoker
EGFR76	81	male	EGFR+	p.Glu746_Ala750del	Ex19del	normal	normal	deleterious	IV	naive	no IBC	smoker
EGFR77	82	female	EGFR+	p.Glu746_Ala750del	Ex19del	normal	normal	normal	IV	naive	no IBC	non-smoker
EGFR78	79	male	EGFR+	p.Glu746_Thr751del	Ex19del	normal	normal	deleterious	IV	naive	no IBC	non-smoker
ALK1	33	male	ALK+	normal		EML4-ALK (E13-A20)	V1	deleterious	IV	naive	no IBC	non-smoker
ALK2	62	male	ALK+	normal		EML4-ALK (E13-A20)	V1	normal	III	naive	no IBC	non-smoker
ALK3	50	female	ALK+	normal		EML4-ALK (E6-A20)	V3	normal	III	naive	no IBC	non-smoker
ALK7	57	female	ALK+	normal		EML4-ALK (E13-A20)	V1	normal	IV	naive	no IBC	non-smoker
ALK8	60	male	ALK+	normal		EML4-ALK (E13-A20)	V1	normal	III	naive	no IBC	non-smoker
ALK14	54	male	ALK+	normal		EML4-ALK (E6-A20)	V3	deleterious	IV	naive	no IBC	non-smoker
ALK16	66	male	ALK+	normal		EML4-ALK (E13-A20)	V1	normal	III	naive	no IBC	non-smoker
ALK17	45	female	ALK+	normal		EML4-ALK (E6-A20)	V3	normal	IV	naive	no IBC	ex-smoker
ALK18	58	female	ALK+	normal		EML4-ALK (E6-A20)	V3	normal	IV	naive	no IBC	unknown
ALK20a	56	male	ALK+	normal		EML4-ALK (E20-A20)	V2	deleterious	III	naive	no IBC	ex-smoker
ALK21	69	female	ALK+	normal		EML4-ALK (E6-A20)	V3	deleterious	IV	naive	no IBC	non-smoker
ALK22	53	male	ALK+	normal		EML4-ALK (E13-A20)	V1	normal	IV	naive	no IBC	smoker
ALK27	63	male	ALK+	normal		EML4-ALK (E13-A20)	V1	normal	IV	naive	no IBC	ex-smoker
ALK29	45	male	ALK+	normal		EML4-ALK (E13-A20)	V1	normal	IV	naive	no IBC	non-smoker
ALK30	57	female	ALK+	normal		EML4-ALK (E6-A20)	V3	deleterious	II	naive	no IBC	ex-smoker
ALK33	64	female	ALK+	normal		EML4-ALK (E13-A20)	V1	normal	IV	naive	no IBC	non-smoker
ALK34	90	male	ALK+	normal		EML4-ALK (E6-A20)	V3	normal	IV	naive	no IBC	non-smoker
ALK36	46	male	ALK+	normal		EML4-ALK (E17-A20)	other	normal	IV	naive	no IBC	non-smoker
ALK37	63	female	ALK+	normal		EML4-ALK (E18-A20)	other	normal	IV	naive	no IBC	non-smoker
ALK38	35	male	ALK+	normal		EML4-ALK (E13-A20)	V1	normal	IV	naive	no IBC	ex-smoker
ALK40	53	male	ALK+	normal		KLC1-ALK (K24-A20)	other	normal	III	naive	no IBC	non-smoker
ALK43	73	female	ALK+	normal		HIP1-ALK (H21-A20)	other	normal	IV	naive	no IBC	ex-smoker
ALK45	66	female	ALK+	normal		EML4-ALK (E13-A20)	V1	normal	IV	naive	no IBC	unknown
ALK46	70	male	ALK+	normal		EML4-ALK (E18-A20)	other	normal	IV	naive	no IBC	unknown
ALK48	59	female	ALK+	normal		EML4-ALK (E13-A20)	V1	normal	IV	naive	no IBC	unknown
ALK50	55	female	ALK+	normal		EML4-ALK (E13-A20)	V1	normal	IV	naive	no IBC	unknown
ALK63	46	male	ALK+	normal		KLC1-ALK (K9A20)	other	deleterious	IV	naive	no IBC	ex-smoker
ALK67	63	female	ALK+	normal		EML4-ALK (E13-A20)	V1	deleterious	IV	naive	no IBC	non-smoker
ALK70	74	female	ALK+	normal		EML4-ALK (E6-A20)	V3	deleterious	III	naive	no IBC	non-smoker
ALK72	67	female	ALK+	normal		EML4-ALK (E6-A20)	V3	deleterious	IV	naive	no IBC	non-smoker
ALK73	56	female	ALK+	normal		EML4-ALK (E13-A20)	V1	normal	IV	naive	no IBC	ex-smoker
