# Supplementary material for: Deciphering the immunosuppressive tumor microenvironment in ALK- and EGFR-positive lung adenocarcinoma
Source: Cancer Immunol Immunother. 2021 Jun 14;71(2):251–65. doi: 10.1007/s00262-021-02981-w (PMC8783861; doi:10.1007/s00262-021-02981-w)
Supplement: Supplementary file 2 — Supplementary file2 (PDF 95 KB) [file 262_2021_2981_MOESM2_ESM.pdf]

Supplement 2: Clinicopathological characteristics of the TCGA cohort comprising 501 lung adenocarcinomas.

**Mutation subtype**	ALK-positive	EGFR-positive	ALK/EGFR-negative
**Number**	5	57	439
**Age:** median (min.- max.)	59 (52 - 74)	66 (45 - 84)	66 (38 - 88)
**sex**			
male	2 (40%)	14 (25%)	212 (48%)
female	3 (60%)	43 (75%)	227 (52%)
**Stage**			
I	4 (80%)	26 (46%)	239 (54%)
II	0 (0%)	15 (26%)	104 (24%)
III	1 (20%)	12 (21%)	68 (15%)
IV	0 (0%)	4 (7%)	21 (5%)
NA	0 (0%)	0 (0%)	7 (2%)
**Prior therapy**			
naive	5 (100%)	57 (100%)	439 (100%)
chemotherapy	0 (0%)	0 (0%)	0 (0%)
