# Supplementary material for: Deciphering the immunosuppressive tumor microenvironment in ALK- and EGFR-positive lung adenocarcinoma
Source: Cancer Immunol Immunother. 2021 Jun 14;71(2):251–65. doi: 10.1007/s00262-021-02981-w (PMC8783861; doi:10.1007/s00262-021-02981-w)
Supplement: Supplementary file 3 — Supplementary file3 (PDF 259 KB) [file 262_2021_2981_MOESM3_ESM.pdf]

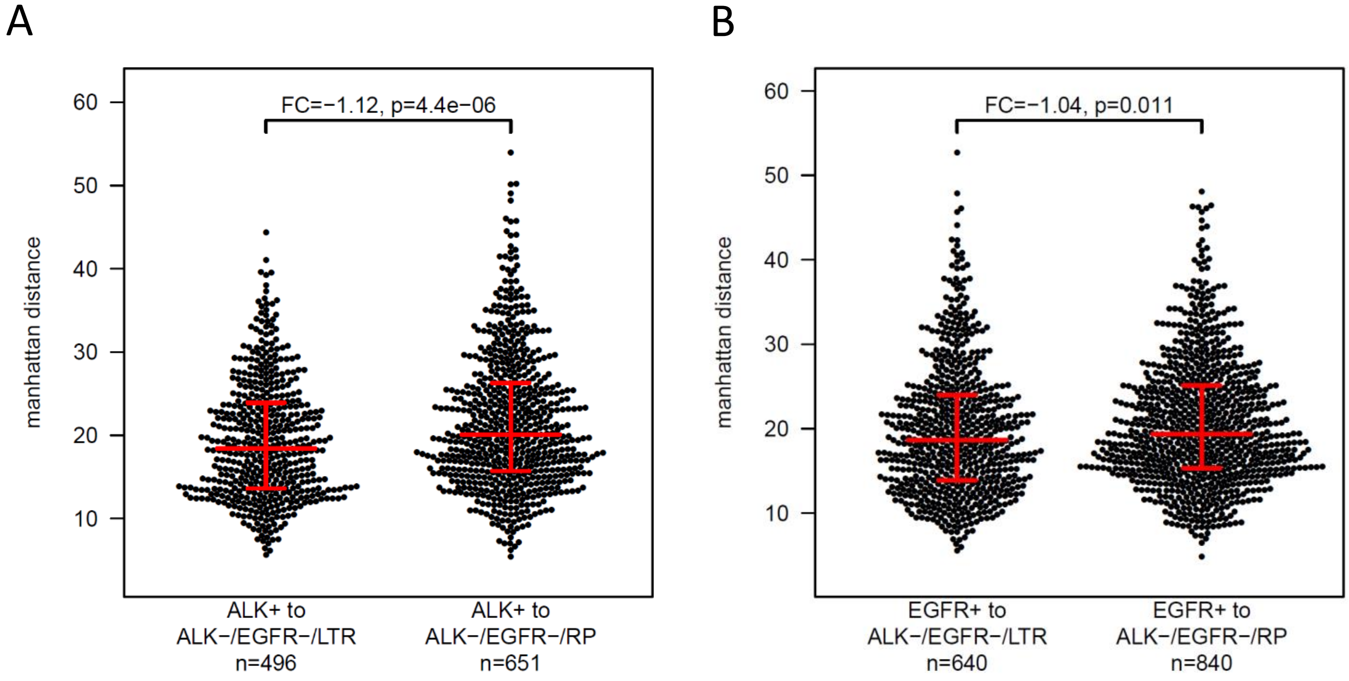

Supplement 3: Analysis of the immunological distance defined as Manhattan distance in the space of 14 immune cell populations. For two tumor subtypes, the immunological distance was calculated for all tumor pairs, i.e. between each tumor of the first subtype and each tumor of the second subtype. **A** ALK-positive tumors were immunologically closer to LTR than to RP ALK/EGFR-negative tumors. **B** EGFR-positive tumors were immunologically closer to LTR than to RP ALK/EGFR-negative tumors.
