# Supplementary material for: Deciphering the immunosuppressive tumor microenvironment in ALK- and EGFR-positive lung adenocarcinoma
Source: Cancer Immunol Immunother. 2021 Jun 14;71(2):251–65. doi: 10.1007/s00262-021-02981-w (PMC8783861; doi:10.1007/s00262-021-02981-w)
Supplement: Supplementary file 4 — Supplementary file4 (PDF 158 KB) [file 262_2021_2981_MOESM4_ESM.pdf]

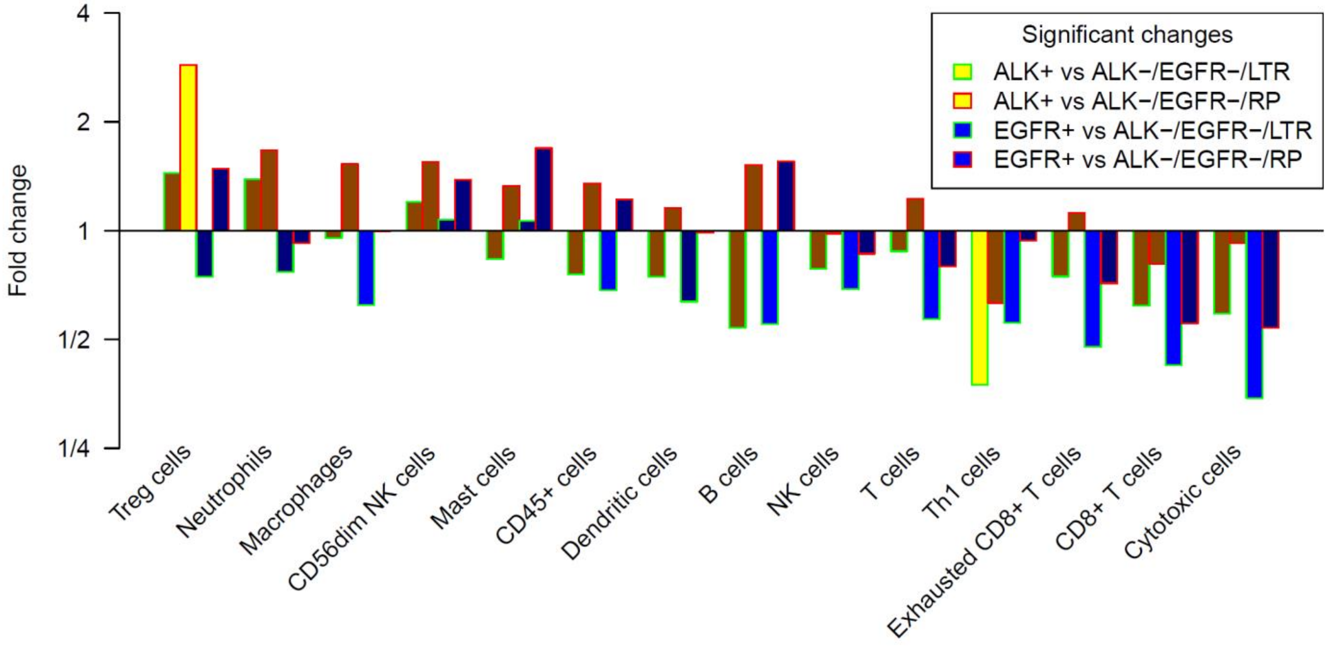

Supplement 4: Comparison of ALK-positive and EGFR-positive cancer with ALK/EGFR-negative cancer that either responded durably to ICB (LTR) or progressed rapidly under ICB (RP). Tregs were significantly higher in AKL-positive cancer compared to RP, Th1 cells were significantly lower in ALK-positive cancer compared LTR. Cytotoxic cells, CD8+ T cells, exhausted CD8+ T cells, Th1 cells, T cells and macrophages were significantly lower in EGFR-positive cancer.
