# Supplementary material for: Deciphering the immunosuppressive tumor microenvironment in ALK- and EGFR-positive lung adenocarcinoma
Source: Cancer Immunol Immunother. 2021 Jun 14;71(2):251–65. doi: 10.1007/s00262-021-02981-w (PMC8783861; doi:10.1007/s00262-021-02981-w)
Supplement: Supplementary file 5 — Supplementary file5 (PDF 422 KB) [file 262_2021_2981_MOESM5_ESM.pdf]

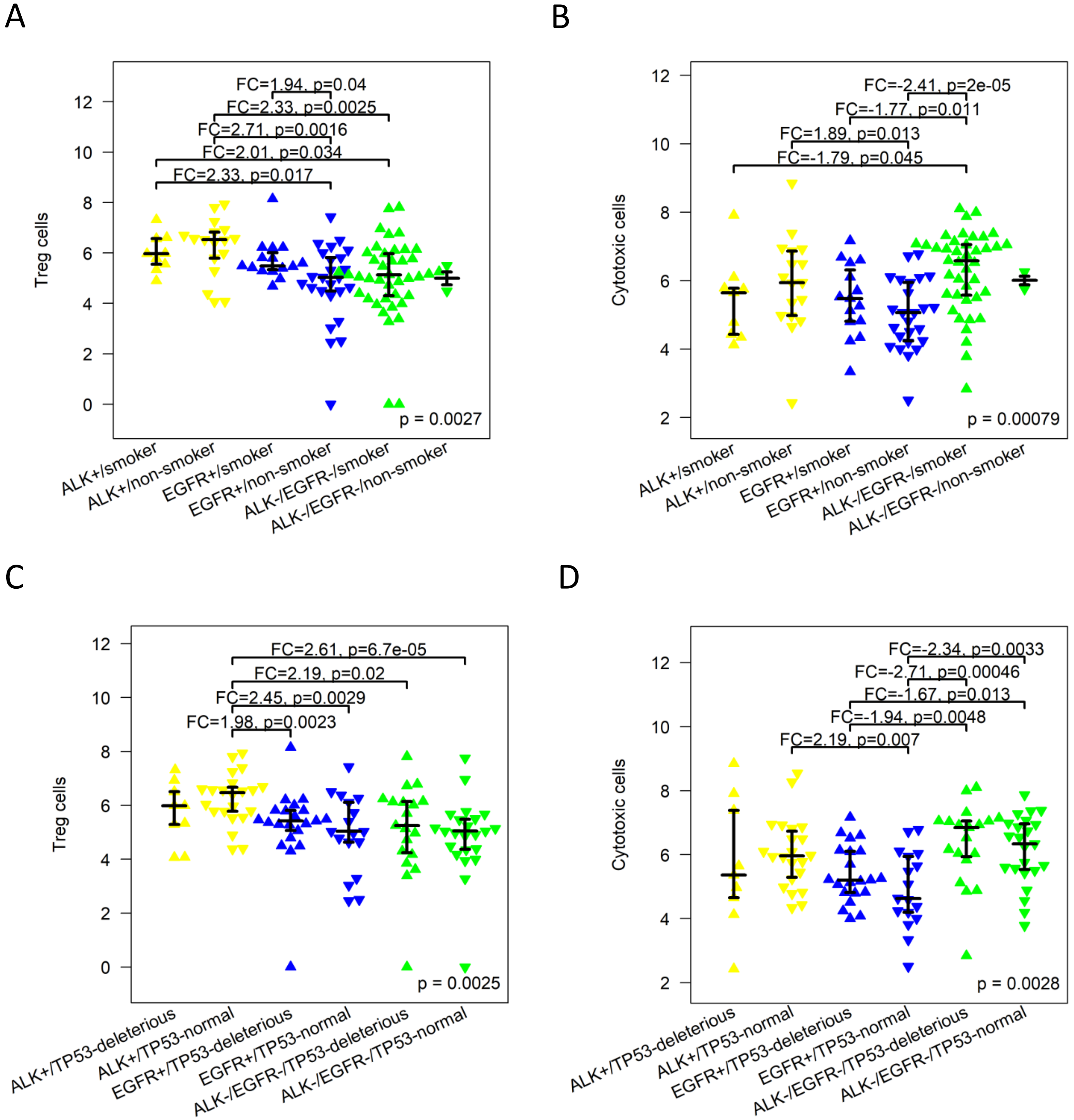

Supplement 5: Analysis of levels of Treg cells and of cytotoxic cells stratified for smoking history (**A/B**) and for TP53 mutation status (**C/D**).
