# Supplementary material for: Deciphering the immunosuppressive tumor microenvironment in ALK- and EGFR-positive lung adenocarcinoma
Source: Cancer Immunol Immunother. 2021 Jun 14;71(2):251–65. doi: 10.1007/s00262-021-02981-w (PMC8783861; doi:10.1007/s00262-021-02981-w)
Supplement: Supplementary file 6 — Supplementary file6 (PDF 682 KB) [file 262_2021_2981_MOESM6_ESM.pdf]

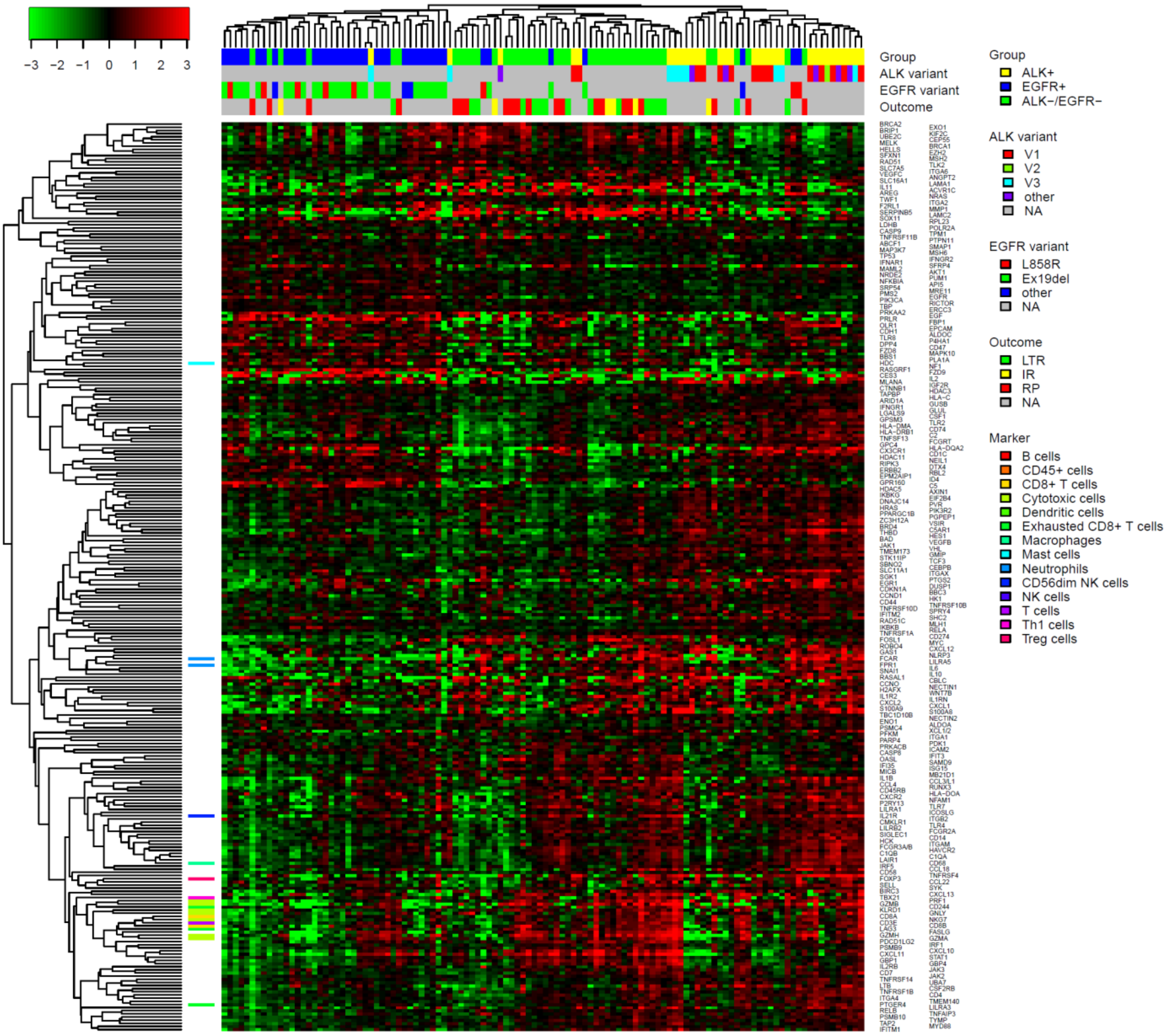

Supplement 6: Heatmap of the 289 genes that were significantly different between ALK-positive, EGFR-positive and ALK/EGFR-negative lung adenocarcinomas (Kruskal-Wallis test, FDR=5%).
