# Supplementary material for: Deciphering the immunosuppressive tumor microenvironment in ALK- and EGFR-positive lung adenocarcinoma
Source: Cancer Immunol Immunother. 2021 Jun 14;71(2):251–65. doi: 10.1007/s00262-021-02981-w (PMC8783861; doi:10.1007/s00262-021-02981-w)
Supplement: Supplementary file 7 — Supplementary file7 (PDF 380 KB) [file 262_2021_2981_MOESM7_ESM.pdf]

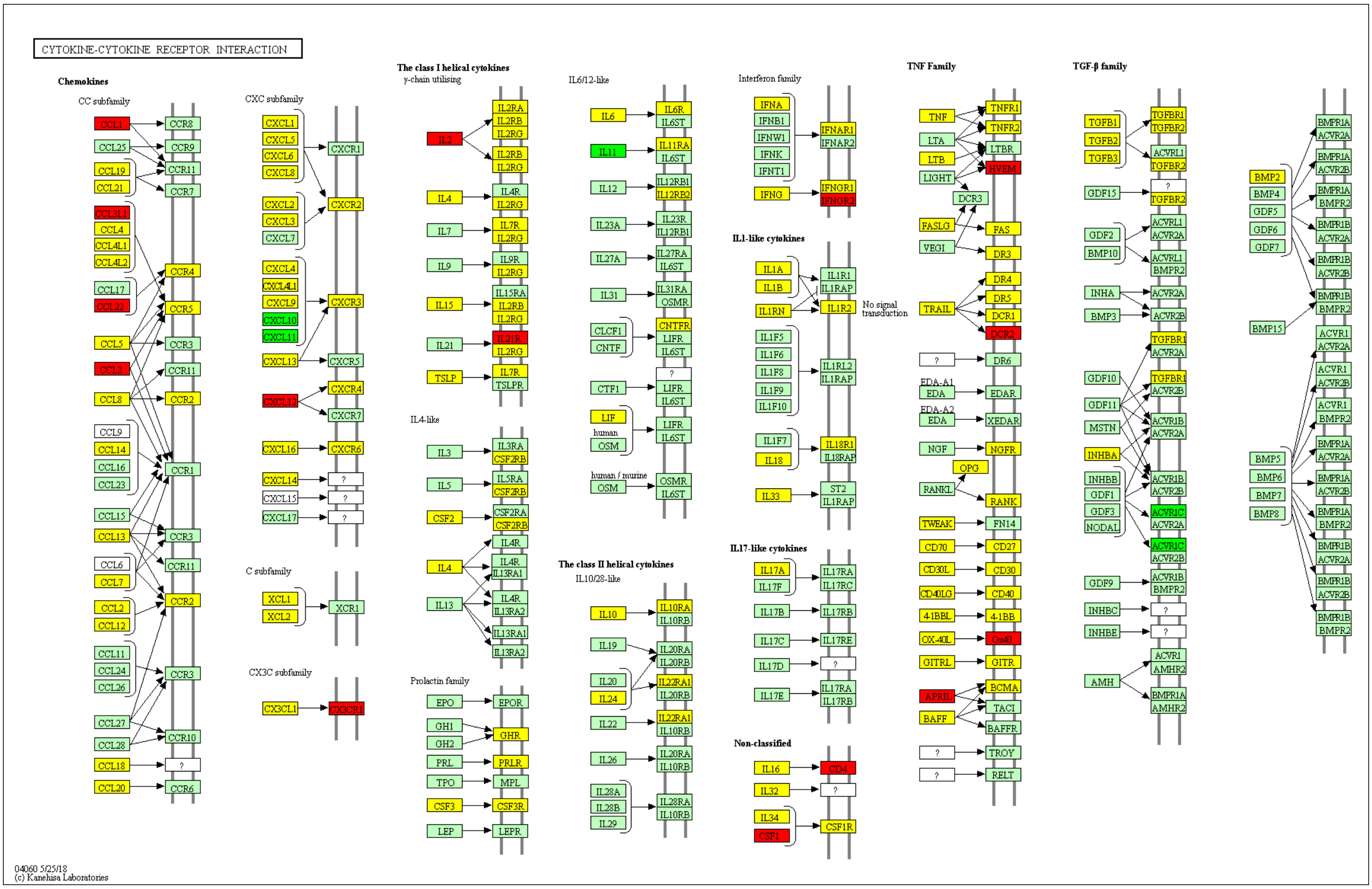

Supplement 7A: Gene expression changes between ALK-positive and ALK/EGFR-negative tumors in the cytokine-cytokine receptor system. Red = overexpression, green = underexpression, yellow = no significant change, light green = human gene, but not covered by the targeted gene expression assay.

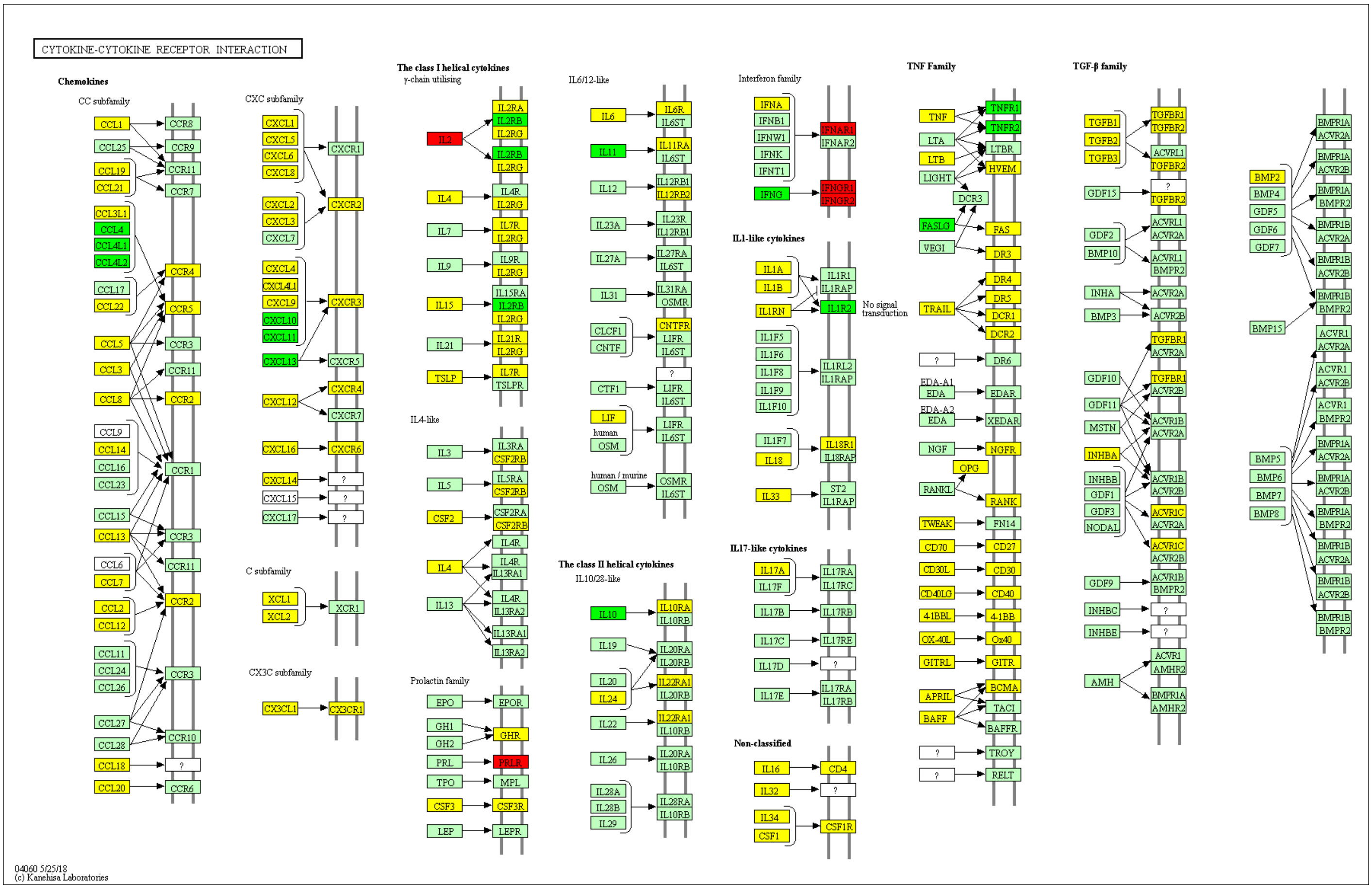

Supplement 7B: Gene expression changes between EGFR-positive and ALK/EGFR-negative tumors in the cytokine-cytokine receptor system. Red = overexpression, green = underexpression, yellow = no significant change, light green = human gene, but not covered by the targeted gene expression assay.

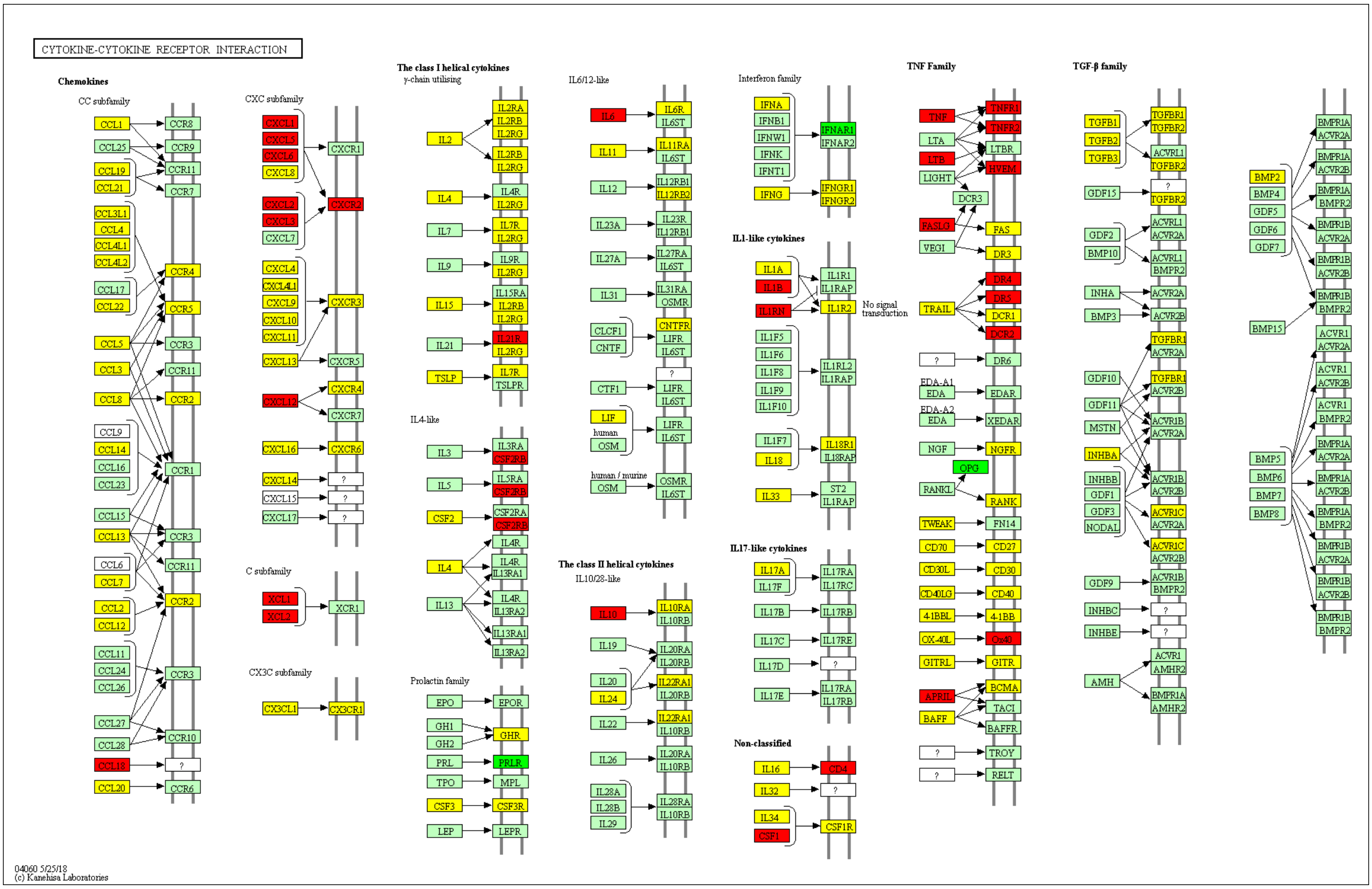

Supplement 7C: Gene expression changes between ALK-positive and EGFR-positive tumors in the cytokine-cytokine receptor system. Red = overexpression, green = underexpression, yellow = no significant change, light green = human gene, but not covered by the targeted gene expression assay.
